# Self-guided transitions and creative idea search: adaptively choosing flexibility versus persistence in divergent and convergent thinking tasks

**DOI:** 10.1098/rsos.241394

**Published:** 2025-03-19

**Authors:** Wilma Koutstaal

**Affiliations:** ^1^ Department of Psychology, University of Minnesota S247 Elliott Hall, 75 East River Road, Minneapolis, MN 55455, USA

**Keywords:** adaptivity, creativity, cognitive flexibility, autonomy, cognitive control, exploration/exploitation

## Abstract

Adaptivity allows individuals to flexibly execute cognitive control to meet dynamically changing task demands while adhering to task goals. Such adaptivity is crucial for navigating complex problem spaces such as creative problem-solving. Recent theoretical and empirical investigations of individuals' tendencies towards flexibility versus persistence have begun to address the questions of how adaptivity may be jointly shaped by general (across-situational) predispositions and by task requirements. However, such investigation is hampered by the lack of comparable ways to quantify trait-like tendencies across different task contexts. Using a Self-Guided Transitions paradigm, in which participants are allowed to autonomously choose whether to continue, to switch or to return to working on either of two concurrently presented problem-solving items, this preregistered study provides evidence for both clear within-individual consistency in the proclivity towards flexibility versus persistence, *and* adaptive modulation of flexibility versus persistence for tasks that predominantly call on divergent versus convergent idea search. Both shifting and dwelling were associated with the generation of more numerous and more original ideas on divergent-thinking tasks—underscoring the creative and ideational rewards to be found *both* by sometimes staying the course (persistence/exploitation) and sometimes choosing to shift our efforts in a different direction (flexibility/exploration).

## 1. Introduction

Creative problem-solving requires both flexibility and persistence to generate novel yet useful and valuable ideas [1–6]. Given the dynamic nature of creative tasks, whether *flexibility* (that is, transitioning to a different item, subgoal or strategy) or *persistence* (continuing with the current item, subgoal or strategy) is optimal for performance often depends on the individual’s current or momentary progress towards task goals and the nature of the task [7,8]. In creative tasks—as well as during cognitive search and goal-directed behaviour more generally—neither stubbornly maintained unyielding persistence (sticking with a current goal, subgoal or stimulus) nor excessive flexibility (distractedly or impulsively continually switching attention to new stimuli, goals or subgoals) is likely to lead to best performance [2,5,9,10]. Rather, effective goal-directed behaviour requires ‘a dynamic, context-dependent balance between maintaining and switching intentions’ [9, p. 343]. This raises two interrelated questions.

The first question focuses on task demands and contextual or task-related adaptivity. To what extent do participants show different degrees of flexibility/persistence depending on the particular tasks or task requirements they are currently facing—that is, *task-related adaptivity*? Do particular features of a given problem-solving or creative search task (e.g. the number and nature of the constraints it involves) modulate the degree to which individuals manifest reduced versus enhanced persistence or flexibility? In particular: are individuals relatively more flexible during *divergent* thinking tasks that are weakly structured, with multiple vaguely defined solution targets that particularly call on varied idea generation and search, but more persistent during relatively well-structured *convergent* thinking tasks with more clearly or narrowly defined solution targets?

The second question focuses on individual (trait-like) differences in within-person consistency of flexibility/persistence across varied problem-solving or search contexts. Alongside, or over-and-above any task-related adaptivity, do some individuals show a trait-like predisposition towards approaching varied problem-solving tasks and cognitive search with a relatively flexible (loosely held) task attentional focus? Do other individuals demonstrate a trait-like predisposition for a comparatively persistent (more tightly held) task focus? Tracking behavioural choices across varied cognitive problem-solving and search tasks, are some individuals characterized by a predominant pattern of flexibility (e.g. frequent transitioning or switching between items or tasks) whereas other individuals show a predominant pattern of persistence (less frequent transitioning and more prolonged phases of continuing working on a given item or task)? Stated simply: how ‘trait-like’ (consistent across contexts and time) is the tendency to be flexible versus persistent?

Despite the fundamental nature of these questions, to date, there is highly mixed empirical evidence regarding whether individuals show an across-task (general) trait-like predisposition towards flexibility versus persistence. There is somewhat stronger, but still mostly indirect, support for task-related adaptivity in flexibility/persistence. Previous studies relating to the existence/nonexistence of a general trait-like characteristic are considered first, in the next section *‘Is there a general (trait-like) predisposition towards flexibility versus persistence?’* A subsequent section, ‘*Task-related adaptivity: Higher flexibility for divergent than convergent tasks?*’, considers findings relating to whether individuals demonstrate heightened flexibility (e.g. show higher switching or exploration) in weakly structured search tasks with vaguely defined solution targets (divergent tasks) compared with well-structured convergent-thinking tasks with clearly defined solutions. A final introductory section provides a brief overview of the current work, which consists of two studies. The first is a pilot study that allowed initial novel experimental tests for both the presence (or not) of a general (trait-like) predisposition towards flexibility/persistence and the extent of task-related adaptivity of flexibility/persistence. These preliminary data formed the foundation for the preregistered methods and hypotheses of the main experiment.

### 1.1. Is there a general (trait-like) predisposition towards flexibility versus persistence?

Two seminal empirical investigations using multiple behavioural search paradigms that notably failed to provide support for a trait-like predisposition towards flexibility versus persistence—despite proposing that there might be such a proclivity—were conducted by Mekern *et al*. [10] and von Helversen *et al*. [11]. Given the centrality of these studies to the hypotheses and methods developed here, they will be described in some detail. Next, studies instead providing initial or suggestive evidential support for the possibility of a (general) trait-like predisposition towards flexibility versus persistence are considered.

Mekern *et al*. [10] examined the persistence/flexibility tradeoff in the context of five different cognitive search tasks, each of which was anticipated to elicit continual switching between persistence and flexibility. The five tasks included a *Word Production Task* (verbal fluency task) that asked participants to generate as many words as possible (in 1 min) that began with a designated letter (L, B or S); an *Alternative Uses Task* [12], where participants were asked to generate as many nontypical/alternative uses as possible (in 5 min) for two common objects (pen, towel); a *Five-Point Design Production Task* (design fluency) where participants were asked to generate as many different designs as possible (in 2 min) connecting multiple instances of five dot arrays by single lines; a *Verbal Search* (Aanagram) task in which participants were tasked to find 30 four-letter words from several six-letter letter sets, with each set presented one at a time, up to a maximum of 14 letter sets; and a *Multi-Armed Bandit Task* in which participants repeatedly chose between one of four slot machine ‘arms,’ where, over 200 trials, the mean payoff for each arm gradually changed, calling upon participants to continuously explore to identify the currently highest paying arm.

For each task, Mekern *et al*. [10] derived clustering and switching scores using specific criteria. In the Word Production Task, consecutive words were identified as part of a cluster if they began with the same two letters, rhymed, differed only by a vowel sound, or were homonyms (based on [13]); in the Alternative Uses Task consecutive responses were identified as part of a cluster if they were related in shape and/or a specific use (e.g. *miniature lighthouse* and *miniature lamp post* for the object *pen*, based on [14]); in the Five-Point Design Production Task consecutive responses were deemed to belong to a cluster if they involved rotation of the design or a part of it, systematic addition or subtraction of lines, or a blended strategy (e.g. [15,16]); in the Verbal Search task, consecutive responses were identified as switches when the participants moved between the letter sets and clusters were identified as the average number of words generated per set; finally, for the Multi-Armed Bandit Task clusters were identified as times when the participant played the same arm for two or more consecutive trials with the average ‘cluster size’ calculated from the second consecutive play and switches defined as the number of switches between arms that were considered exploratory (e.g. in the series of plays, arm1-arm1-arm1-arm2-arm1 only the initial switch from arm1 to arm2 was considered exploratory).

Comparing the measures of switching and clustering within individuals across these varied tasks, Mekern and colleagues [10] found very little evidence for across-task consistency, that is, little evidence for interindividual (trait-like) consistency in flexibility or persistence. Specifically, out of 10 possible pairs of tasks, they found that clustering scores did not significantly correlate between any two tasks. And out of 10 possible pairs, switching positively correlated for only one pair: the Alternative Uses Task and the Five-Point Design Production Task, arguably the two most divergent thinking tasks among the included tasks (Spearman’s *r* = .344, 95% CI = [.18, .49], *p* < .001, Holm-corrected for multiple comparisons).

These results appear to strongly argue against any across-task (general) trait-like predisposition towards flexibility versus persistence. Yet it is possible that the considerable variation in how, precisely, clustering (persistence) and switching (flexibility) were operationalized for the various tasks obscured detection of interindividual (trait-like) consistency across the tasks. Additionally, as Mekern *et al*. [10] used after-the-fact researcher-based criteria to determine whether a participant’s responses belonged to the same or a different category or response cluster, it is not known to what extent the researcher-defined clustering/switching corresponds to a participant’s actual (in the moment) thinking or search processes. Any such misalignment between the participant’s thinking and the researcher-imposed clustering/switching rubric might further obscure any signal of within-individual (trait-like) consistency in persistence or flexibility between the tasks.

Nonetheless, a study by von Helversen *et al*. [11] that used three different exploration/exploitation behavioural paradigms—but now with dependent outcome measures that were closely tied to participants' objective behavioural performance—likewise failed to find evidence for an across-task (general) trait-like predisposition towards flexibility versus persistence. The three behavioural tasks adopted by von Helversen [11] included: (i) a foraging task requiring sequential search (foraging for fish, [17]), with exploration assessed by the number of ponds visited; (ii) a sequential choice task requiring choosing a candidate from a pool of applicants (a version of the secretary task, [18]), with exploration assessed by the mean percentage of options that the participant considered before accepting an option; and (iii) a multi-armed bandit task involving repeatedly choosing from a set of five options accompanied by varied payoffs but where the arms had constant payoffs within each block of trials (e.g. [19]), with exploration assessed by the percentage of choices that involved a switch between options. Each task was administered in two versions (a short and long version).

Structural equation modelling by von Helversen *et al*. [11] failed to yield support for a single-factor model underlying exploratory behaviour in the three tasks. Rather, the best fitting model was a three-factor latent variable model, where the latent factors were correlated—suggesting that exploration is task specific, but there is common variance shared across tasks. Indeed, there even appeared to be a modest negative relation between exploration in the bandit task and the fishing task, and between exploration in the bandit and the secretary task. Notably, these results were obtained even though, as expected, there were significant positive correlations between the two short and long versions of each of the *same* tasks also included in the study (*within-task* correlations of .82, .95 and .62 for the fishing, bandit and secretary tasks, respectively).

On the one hand, the failure to observe a generalized (across-task) factor underlying exploration in the study by von Helversen *et al*. [11] counts as further evidence against domain-general (trait-like) preferences for exploration–exploitation. On the other hand, despite the use of measures that were closely tied to participants' objective behavioural performance, it is possible that marked variation in the choice and other parameters across the three different tasks may have overshadowed the ability to observe any consistent individual tendencies. The tasks differed in whether participants chose between the same options versus different options over time (e.g. the bandit task versus the secretary task), and whether participants were limited by the amount of time they had versus the number of choices they could make (e.g. foraging for fish versus the bandit task). Critically, the tasks also included notable variation in the primary outcome measures (the number of ponds visited versus the proportion of options searched versus the proportion of switches).

By contrast, other research has provided some evidential support for generalized (across-task) trait-like consistency in exploration tendencies. Hills *et al*. [20] found that participants who explored more in space in an external spatial-foraging task (finding ‘resource’ pixels in a two-dimensional maze) also explored more in a lexical Scrabble-like task, such that those who were more wide-ranging spatial explorers also more frequently chose to move to the next of several different letter sets. That is, exploratory behaviour in the spatial-foraging task was a significant predictor of letter-set time. In further work, Hills *et al*. [21,22] (but see also [23] for counter-evidence) observed an across-task transfer effect between the spatial foraging and lexical word-finding tasks. Participants in the spatial foraging task who earlier had experienced clustered visual search, where the ‘pixel resources’ they found were spatially nearby to each other, subsequently, in the lexical task, stayed longer (persisted) with each letter set significantly more than did participants who earlier encountered diffuse (widely dispersed) resources during the spatial foraging task. The authors propose that these results, together with other findings, provide theoretical support for a generalized cognitive search process such that ‘search behaviors in different domains use a shared underlying neural architecture’ [21, p. 604]. If there are shared cognitive parameters across varied search tasks then, depending on how those parameters are initially set or modulated (e.g. not only by recent experiences but also by pre-existing trait-like individual differences), then these outcomes may be viewed as aligned with (or at least not in conflict with) within-individual (trait-like) consistency in exploration/flexibility versus exploitation/persistence across tasks or domains.

Within-individual consistency in the frequency of shifting also might be expected from empirical evidence from what is known as the ‘free concurrent dual-tasking paradigm’ [24]. In this paradigm, two different tasks (A and B) are always concurrently visible. The participant is entirely free to choose when to respond to each task provided that, overall, they treat both tasks as equally important, and aim to maximize their performance on both tasks within the allotted time. For instance, a participant might be asked to classify individually presented letter-number stimuli (e.g. *M2, E5*) according to either a digit classification rule (*was the digit odd or even?*) or a letter classification rule (*was the letter a consonant or a vowel?*).

Findings from the free concurrent dual-tasking paradigm have provided evidence for strong within-individual preferences for how participants tend to organize their responses to the two tasks. In particular, individuals generally tend to demonstrate patterns of responding that fall into one of three response types: (i) long sequences of working on one task before switching (blocking), (ii) switching repeatedly after relatively short sequences (switching or alternating), or (iii) a pattern of responding temporally close in time to both tasks (termed response grouping). These preferred response strategies have been observed across different dual-task combinations and studies [24–27] and appear to reflect ‘a preference for more structured versus flexible ways to cope with concurrent tasks’ [25, p. 1428]. Notably, these free concurrent dual task paradigm findings involve highly similar measures of participants' autonomous choices (to stay or to switch) across the tasks—unlike the varied outcome measures used by Mekern *et al*. [10] and von Helversen *et al*. [11].

More recently, aiming to more fully characterize the contributions of flexibility and persistence to creative performance, Wu & Koutstaal [5,28] introduced a ‘Self-Guided Transitions’ (SGT) paradigm that is broadly similar to the free concurrent dual-tasking paradigm but with two essential differences. First, in the SGT paradigm, rather than participants being asked to concurrently choose which of two tasks to perform, where each task has different goals and response rules, participants are given two *items* or prompts for the *same task* (with the same rules, goals, etc.), and are free to choose when, and for how long, to work on each of two concurrently presented *stimulus items* for that same task. Second, in the SGT paradigm there is no instructional requirement for participants to attempt to devote equal attention or effort to the two items. Within the designated time period (e.g. 6 min) participants are entirely free to choose whether to concentrate their efforts on either one of the two items, continuing to work on each as long as they wish, before switching to the other, or back again.

The new process-based method of operationalizing flexibility and persistence involves assessing the number of autonomously chosen ‘self-guided transitions’ (SGTs) that participants make between the two concurrently available task items [5]. In this approach, flexibility is indexed by the number of times that participants explicitly choose to shift from working on one of the two possible task items to the other item, or back again (‘shift-count’). Persistence is indexed by the average number of responses participants produce each time they choose to work on a given item (‘dwell-length’).

**Figure 1.**
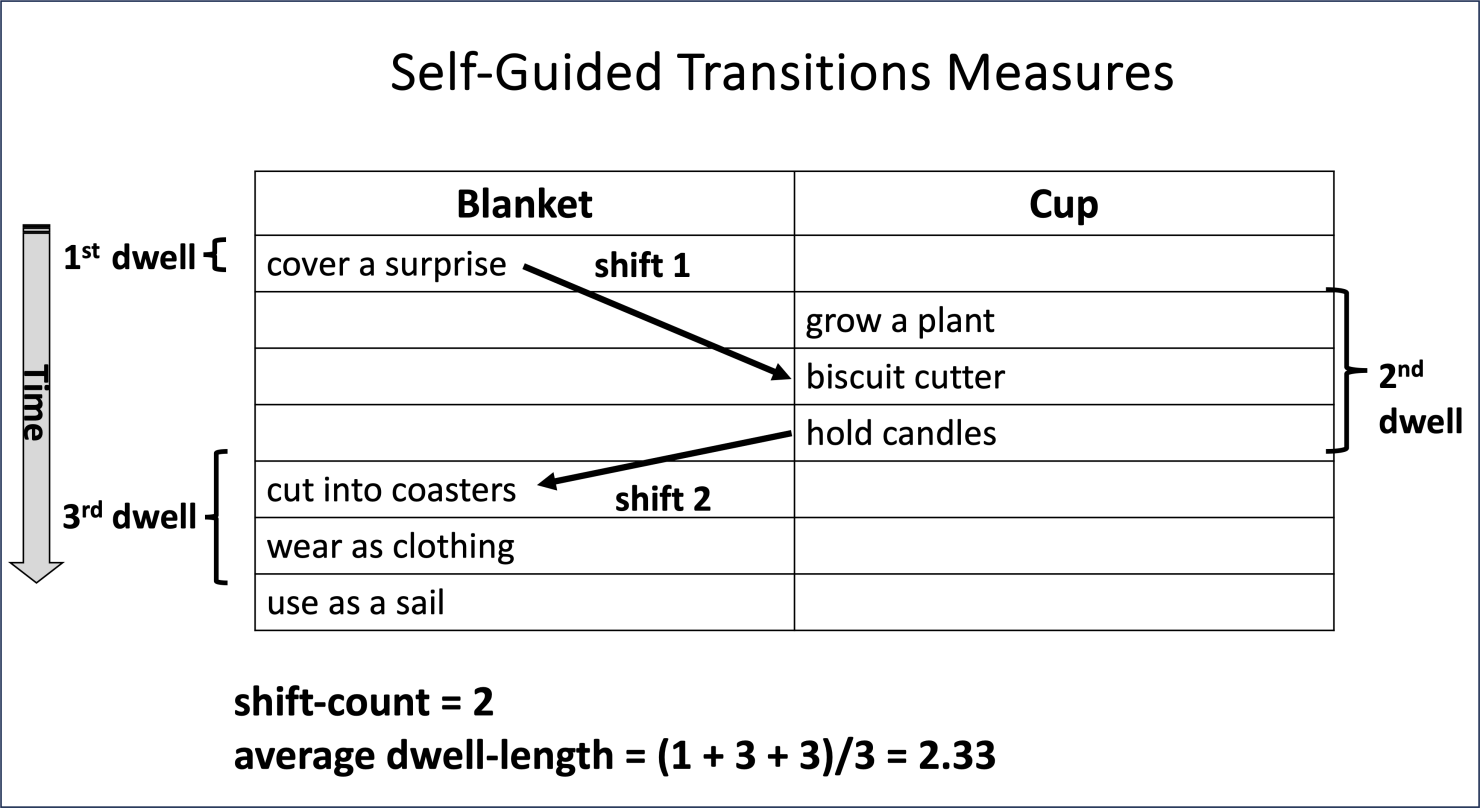
Illustration of how shift-count and dwell-length are calculated. Shift-count and dwell-length are retrospectively coded from computer screen-recordings of the participants' in-the-moment responses as they autonomously choose to work on one or the other of the two provided items (in this case, for the Alternative Uses Task, requiring participants to creatively generate nontypical possible uses for the objects of ‘*blanket*’ and ‘*cup*’).

Figure 1 provides an illustration of how shift-count and dwell-length are calculated, in this case for stimuli from the Alternative Uses Task.

Importantly, this process-based measure of a participant's choices to focus on one or the other of the two provided items does not depend on the specific *content* of participants' responses to the task at hand. Thus this approach allows the implementation of various problem-solving tasks with varying degrees of task constraints, and yields measures of flexibility and persistence that are more directly comparable across different tasks. The SGT paradigm can thus provide a clearer measure of both task-related adaptivity and an individual’s across-task propensities in favour of either flexibility or persistence.

From a theoretical perspective, Self-Guided Transitions also offer the opportunity to more clearly decouple a participant’s voluntary and conscious behavioural strategy choices during a task from other (inferred and possibly subconscious) cognitive processes that might contribute to an individual’s problem-solving capabilities. During the cognitive search tasks used in Mekern *et al*. [10]—that adopted varying measures of switching and clustering for different tasks—participants were not aware of the researcher-defined categories and might not systematically search in those researcher-defined categories. Therefore, the flexibility and persistence scores may reflect neurocognitive features, such as the speed of automatic spreading activation in semantic networks [29–31], that were not consciously controlled by the individual. The new shift-count and dwell-length measures offer a unique opportunity to quantify the autonomous aspect of individuals' search in terms of their conscious choices to either switch to work on a different task item or, instead, choosing to continue working on the same task item.

The SGT paradigm was first introduced by Wu & Koutstaal [5] as a behavioural measure of cognitive flexibility and creativity-related adaptivity. They demonstrated that, for the Alternative Uses Task, both shift-count and dwell-length explained a significant proportion of variance in measures of fluency and originality on a composite measure of three independently assessed creative tasks—providing strong support for the dual-process model of creativity [2] according to which creative problem-solving requires both flexibility and persistence to generate novel yet useful and valuable ideas. This initial study included several additional creative tasks, including a more complex and naturalistic ‘Garden Design’ task [32], administered with a think-aloud protocol and where participants provided concurrent sketches of their evolving garden. Given this extensive participant testing protocol, Wu & Koutstaal [5] incorporated only two SGT tasks: a predominantly convergent Anagram problem-solving task and the largely divergent Alternative Uses Task. In line with an across-task (trait-like) individual tendency towards persistence, the study found that dwell-length was significantly positively correlated across the AUT and Anagram tasks (*r* = .31). However, there was only a weak and nonsignificant correlation of shift-count between AUT and Anagram (*r* = .11), though shift-count was predictive of a composite measure of overall quality on the Garden Design task.

**Figure 2.**
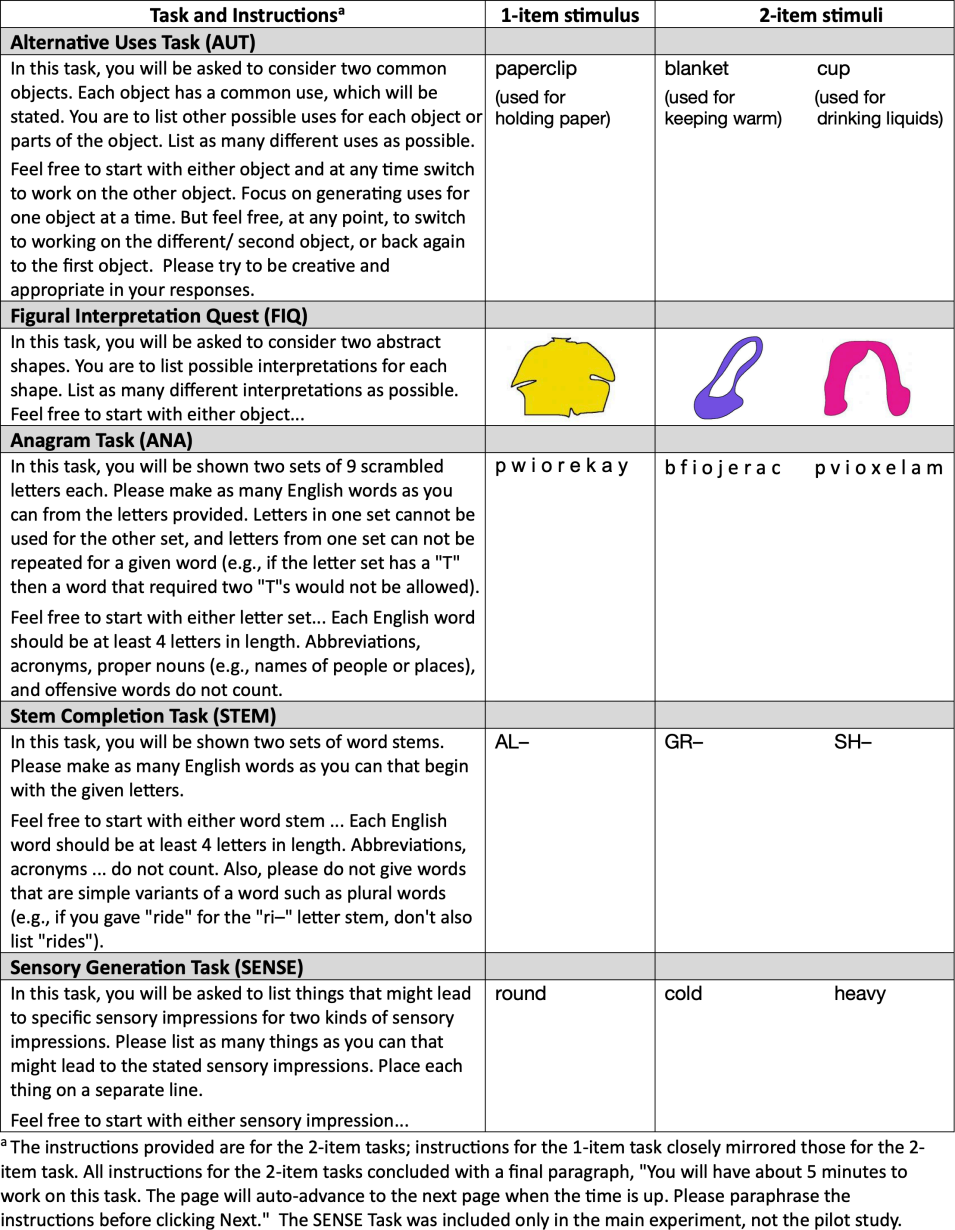
Stimuli and instructions for the Self-Guided Transition tasks.

In a subsequent study, Wu & Koutstaal [28,33] incorporated an expanded set of four SGT tasks. Besides the Anagram and Alternative Uses Task, there was also a more perceptually prompted divergent thinking task (the Figural Interpretation Quest) and a Word Stem Completion Task. In the Figural Interpretation Quest, participants were shown ambiguous coloured shapes, one at a time, and asked to generate as many different interpretations for the shape as possible [34,35]; see figure 2 for example stimuli. In the Word Stem Completion Task, participants were asked to generate English words, each composed of four or more letters, that might complete provided two-letter word beginnings or stems (e.g. *AL—*might be completed with *alternate, aloft, alleviate*).

The study of Wu & Koutstaal [28] included a between-subjects instructional manipulation of whether participants were directed to regularly switch between the items (required regular-switch condition), were encouraged to continue working on each item until they were no longer actively finding ideas for that item (asked-to-stay condition), or were allowed to naturally choose if and when to switch between the two task items (free-to-choose condition). The across-task correlations between shift-count and dwell-length in the free-to-choose condition were not the focus of the earlier study, but were newly analysed as part of the pre-registration for the current study. These new analyses are reported here (see Pilot Study). To briefly presage the outcomes of those analyses, the preliminary data provided strong evidence for within-individual across-task (trait-like) consistency of both flexibility (assessed by shift-count) and, to a somewhat lesser extent, across-task persistence (assessed by dwell-length).

### 1.2. Task-related adaptivity: higher flexibility for divergent than convergent tasks?

Several considerations suggest that (predominantly) divergent thinking tasks may be associated with higher flexibility and switching than are (predominantly) convergent thinking tasks. At a broad level, divergent thinking has long been linked with an ability to flexibly switch between different conceptual and perceptual perspectives, categories, sets or approaches [2,6,12,14,30,31,34,36]. Flexibility is often necessary when the search target is only vaguely or indeterminately specified and the required cognitive operations or ‘mental route’ to take in order to find potential targets is uncertain and unknown. By contrast, convergent tasks, such as the Anagram problem-solving task, provide what Cohen *et al*. [37, p. 940] characterized as ‘a well-structured [...] versus unknown and unpredictable environment.’ In an Anagram task, for example, the letter set stimuli are continually visually present, the task rules are explicitly stated (e.g. generate English words using the provided letters), and the search targets are familiar and readily identified (e.g. English words that are at least four letters in length, that are not abbreviations, acronyms, proper nouns or offensive words). These well-structured conditions might elicit a form of systematic exploration, or what Cohen *et al*. [37] refer to as ‘controlled exploration’ involving a fair degree of top-down cognitive control and somewhat extended periods of focused attention (persistence) on a given letter set.

By contrast, in divergent tasks, such as the Alternative Uses Task (AUT), in which participants are presented the name of a common everyday object such as ‘*cup*’ and asked to generate as many nontypical/alternative uses for the object as possible, the search targets are quite vaguely specified with many, often only weakly interrelated, alternatives. Divergent thinking tasks thus may benefit from a comparatively looser degree of top-down cognitive control [38,39] and enhanced receptivity to low-level perceptual data such as shapes, sounds or visuomotor information [38]. For the AUT, a somewhat relaxed form of top-down control and the more vaguely specified search target might elicit comparatively briefer periods of focused attention on a given object, with more frequent transitions from one of the two provided objects to the other, and back again (higher flexibility).

In line with this suggestion, other research has found that promoting more frequent item switching was beneficial to divergent thinking performance [40,41]. For instance, using a category exemplar-generation task, where participants are asked to list possible instances or examples within a given indicated category (e.g. *cold things*), Smith *et al*. [42] found that requiring participants to repeatedly alternate between two concurrently presented items led to the production of a higher number of exemplars, and to some degree also more novel responses, than if participants were instructed to work on each item for a continuous (blocked) period of time. However, this alternation benefit was observed only for comparatively loosely defined (more open-ended) categories, such as a*d hoc* categories (e.g. *equipment you take camping*) or sense impression categories (e.g. *cold things, heavy things*) rather than more structured taxonomic categories (e.g. *clothing, birds*).

Suggestive findings linking increased frequency of shifting to divergent thinking are also provided by a recent study in which participants completed both separately administered measures of divergent and convergent thinking and two versions of a multi-armed bandit task [43]. On an equal-solution four-armed bandit task that did not have a ‘best’ button (so the same score could be achieved with any of the buttons), participants with a high score on divergent thinking switched between buttons significantly more often than did other participants on all five successive periods of the task. High divergent-thinking scoring participants also switched significantly more often during the first few periods of a more challenging eight-armed bandit task that did have a ‘best button;’ that is, high divergent-thinking scoring participants also switched more frequently during the initial exploratory phase when the best button had not yet been identified. By contrast, participants' *convergent* thinking scores were not consistently related to the number of switches in either version of the bandit task, but were significantly related to the number of clicks specifically on the *best button* during the final period of the best button task—that is, after the optimal solution had been identified.

Findings on the benefits of task switching in relation to another predominantly convergent thinking task—the Remote Associates Task—likewise are mixed at best. In the Remote Associates Task, participants are presented sets of three apparently unrelated words, for which they are challenged to find a fourth word that can be combined with each of the three stimulus words (e.g. the words *dew/comb/bee* can all be combined with the word *honey: honey dew, honey comb* and *honey bee*). Some studies using the Remote Associates Task have provided support for the benefits of increased frequency of switching between items, compared with a continuous item focus, on the generation of correct solutions [41]. However, other studies have failed to find a benefit of heightened switching on solution rates for this convergent task [44] (Study 2) or have found a benefit only for a subset of RAT stimuli with a common misleading associate [45]. For example, for the three words *stick, point and maker*, the word *pin* is a misleading common associate because it is associated with both *stick (stickpin*) and *point (pinpoint*) but not with *maker,* whereas the correct solution is instead *match* (*matchstick, match point and match maker*). Indeed, not only did Sio *et al*. [44] (Study 1) fail to find a benefit of required switching on this convergent task, but they found a significant *negative* effect of task-switching on RAT problem-solving accuracy.

More recently, using a type of spatial-foraging priming paradigm, Malaie *et al*. [46] asked participants to find targets on a map under one of two spatial conditions invoking either divergent goals or a single goal. In the divergent-goals condition, participants were asked to draw a route from a fixed originating point to each of five different (dispersed) target locations. By contrast, in the single-goal condition participants were asked to draw five routes, each of which converged on the same target location. Participants in the divergent-goals condition subsequently generated significantly more correct responses per trial on a relatively loosely structured word stem completion task (e.g. generating English words that begin with the letters *AB– or DA–*) than did participants in the single-target condition. By contrast, performance of participants in the single goal condition significantly surpassed that of the divergent-goals condition on the more structured, tightly constrained Remote Associates Task. These findings may suggest a possible causal link between external/perceptual-motor search strategies and internal cognitive search (cf. [21]). Equally important, the *differential patterns* of prior divergent-goal versus single-goal spatial search on subsequent performance suggest that *the form of internal cognitive search—*divergent versus convergent—may differentially benefit from, respectively, heightened exploration/flexibility versus bolstered exploitation/persistence.

### 1.3. Overview of the current studies

The current report presents (i) preliminary data analyses from the free-to-choose condition from the experiment by Wu & Koutstaal [28,33], including new analyses and comparisons of the across-task correlations between shift-count and dwell-length, and (ii) a preregistered study with an extended participant sample. Given that the preliminary data served as the basis for the specific preregistered methods and hypotheses on which the main study were based, the hypotheses are provided after the pilot data and after an overview of the main experiment are presented.

## 2. Pilot study

The preliminary data are based, as noted, on new analyses of the free-to-choose experimental condition of Wu & Koutstaal [28]. In this condition (*n* = 49, 31 female, 18 male, average age 20.04 years, s.d. = 2.66) undergraduate participants took part in four SGT idea-generation tasks, described next.

### 2.1. Procedure

Two of the four SGT tasks were divergent-thinking tasks. See figure 2 for specific examples of the stimuli and the 2-item instructions for each of the tasks. One of the divergent thinking tasks was the predominantly conceptually prompted Alternative Uses Task (AUT) during which participants were asked to generate nontypical uses for a common object (e.g. *cup*) and the typical or customary use was stated (e.g. *used for drinking liquids*). The second divergent thinking task was the perceptually prompted Figural Interpretation Quest (FIQ) [34,35], during which participants were shown ambiguous coloured shapes, one at a time, and were asked to generate as many different interpretations for the shape as possible. For instance, the ambiguous purple shape in figure 2 might variously be interpreted as a ballet shoe, a purse, a hair-tie or an island. Although the perceptually prompted FIQ is a more recently developed task, that is less extensively studied than the AUT, it has been shown to significantly positively correlate with other divergent-thinking tasks. Specifically, FIQ fluency, flexibility and originality scores positively correlate with corresponding performance on predominantly conceptual tasks such as the AUT and a modified version of the Torrance ‘Just Suppose’ [47] task. FIQ scores also positively correlate with performance on another (more abstract) perceptually prompted divergent-thinking task (the Pattern Meanings Task) [48] and with performance on a design product ideation task, in which participants were asked to generate varied creative ideas for urban gardening or ideas for picnics [34,49].

The other two SGT tasks were convergent-thinking tasks, including the highly structured Anagram task (generate English words that are at least four letters in length using the letters provided in a set of nine letters, such as *b f i o j e r a c*, excluding abbreviations, acronyms, etc.) and a somewhat less tightly structured Stem Completion task (generate English words that are at least four letters in length that begin with the specified two letters, such as *AL–* or *GR–*, excluding abbreviations, acronyms, etc).

Crucially, while introducing this heterogeneity in the nature of the tasks to assess how generalizable the findings were across comparatively unconstrained versus constrained task contexts, we held constant numerous other aspects of the tasks, including the task duration, stimulus display format, response input method, and—most notably—the operationalization of the measures of flexibility (shift-count) and persistence (dwell-length). (See figure 1 for an illustration of the calculation of these SGT measures for the AUT task. The display format was highly similar for each of the other tasks.)

The four SGT tasks were administered in an interleaved order alternating between divergent and convergent thinking tasks, specifically: Alternative Uses Task, then Anagram, then Figural Interpretation Quest, then Stem Completion. To familiarize participants with the response requirements, each task type (AUT, ANA, FIQ, STEM) was first administered with only a single stimulus (1-item format) for 3 min, followed directly by the 2-item format for 6 min. For each 2-item task, in addition to being reminded of the instructions specific to the task, participants were instructed that they were free to start with either item (object, letter set, word stem) and at any time switch to work on the other item. Participants were asked to focus on generating ideas for one item at a time, but to feel free, at any point, to switch to working on the different/second item, or back again to the first object.

### 2.2. Results

Table 1 provides descriptive statistics for correct responses (fluency) on the four tasks, including fluency scores for both the 1-item and 2-item format. Table 2 provides descriptive statistics for the SGT process measures (shift-count and dwell-length) for the 2-item tasks. Additionally, given that the tasks varied in the number of correct responses, we also calculated a proportion shift-count measure (that is, shift-count divided by the number of correct responses); descriptive statistics for the proportion shift-count measure can be found in table 3.

**Table 1.** Descriptive statistics for fluency on the 1-item and 2-item tasks (Pilot Data).

task	mean	lower 95% CI	upper 95% CI	median	std. dev.	*N*
*one item*						
AUT	7.30	6.31	8.28	6.50	3.44	49
FIQ	9.37	8.10	10.63	9.00	4.41	49
ANA	5.71	4.78	6.64	6.00	3.24	49
STEM	9.62	8.27	10.97	9.00	4.69	49
*two item^a^*						
AUT	16.96	15.66	18.27	16.62	4.50	48
FIQ	20.93	18.53	23.34	19.21	8.28	48
ANA	16.97	15.25	18.70	16.80	5.95	48
STEM	29.73	25.88	33.59	24.80	13.27	48

AUT, Alternative Uses Task; FIQ, Figural Interpretation Quest; ANA, Anagram Task; STEM, Stem Completion Task.

^a^ Two item values are based on the total fluency for the two items.

**Table 2.** Descriptive statistics for the SGT process measures for the 2-item tasks (Pilot Data).

task and measure	mean	lower 95% CI	upper 95% CI	median	std. dev.	*N*	*N* outliers (IDs^a^)
AUT shift-count	5.81	5.08	6.55	6.00	2.53	48	—
AUT dwell-length	2.67	2.29	3.05	2.46	1.30	48	—
FIQ shift-count	7.96	6.59	9.33	7.00	4.71	48	—
FIQ dwell-length	2.51	2.16	2.85	2.37	1.18	48	—
ANA shift-count	4.50	3.65	5.35	4.00	2.93	48	1 (215)
ANA dwell-length	3.99	3.22	4.76	3.41	2.65	48	1 (323)
STEM shift-count	6.10	4.18	8.03	4.00	6.62	48	2 (215, 290)
STEM dwell-length	8.76	6.72	10.81	7.33	7.05	48	1 (323)

AUT, Alternative Uses Task; FIQ, Figural Interpretation Quest; ANA, Anagram Task; STEM, Stem Completion Task.

^a^ IDs, participant identification numbers for outlier values; all outlier values were higher than the mean for the designated measure. Note that outlier values are not errors—and, indeed, individual differences or variation in the measures of dwell-length and shift-count are of primary research interest. Thus, the descriptive statistics are calculated including all participants, but the median is also provided; additionally, all subsequent correlational analyses report both Pearson’s correlation and Spearman’s nonparametric rank correlation coefficient (*rho),* which is less influenced by outlier values.

**Table 3.** Descriptive statistics for the proportion shift-count SGT measures for the 2-item tasks (Pilot Data).

task	mean	lower 95% CI	upper 95% CI	median	std. dev.	*N*	*N outliers (IDs* ^*a*^ *)*
AUT proportion shift	.67	.62	.72	.71	.18	48	—
FIQ proportion shift	.71	.66	.76	.75	.18	48	—
ANA proportion shift	.52	.47	.58	.52	.20	48	—
STEM proportion shift	.41	.33	.49	.38	.27	48	—

AUT, Alternative Uses Task; FIQ, Figural Interpretation Quest; ANA, Anagram Task; STEM, Stem Completion Task.

^a^ IDs, participant identification numbers for outlier values; all outlier values were higher than the mean for the given measure.

To address the first key research question of whether participants showed across-task (trait-like) consistency in flexibility or persistence, we calculated the across-task correlations of shift-count, dwell-length and proportion shift-count (see tables 4–6, respectively). These analyses provided strong evidence of within-individual across-task (trait-like) consistency. In particular, as can be seen from table 4, five out of the six pairwise correlations for shift-count were significant. Across all six pairs, the average correlation (based on Fisher *Zr* transformation) was *r* = .55. We observed a similar numerical but less strong pattern of positive intercorrelations for dwell-lengths, with significant correlations between Stem and Anagram dwell-lengths (*r* = .66) and Stem and FIQ dwell-lengths (*r* = .33). Across all tasks, the average pairwise correlation (based on Fisher *Zr* transformation) for dwell-lengths was *r* = .30.

**Table 4.** Across-task correlations for shift-count on the 2-item tasks (Pilot Data).

task	AUT	FIQ	ANA	STEM
AUT	—			
FIQ	.55*** [.63***]	—		
ANA	.35* [.48***]	.49*** [.56***]	—	
STEM	.28^ [.44**]	.54*** [.54***]	.72*** [.51***]	—

AUT, Alternative Uses Task; FIQ, Figural Interpretation Quest; ANA, Anagram Task; STEM, Stem Completion Task. Spearman’s *rho* in square brackets. ***p < .001,**p < .01,*p < .05, ^*p* < .10

**Table 5.** Across-task correlations for dwell-length on the 2-item tasks (Pilot Data).

task	AUT	FIQ	ANA	STEM
AUT	—			
FIQ	.26^ [.25^]	—		
ANA	.16 [.39**]	.12 [.21]	—	
STEM	.11 [.29*]	.33* [.53***]	.66*** [.40**]	—

AUT, Alternative Uses Task; FIQ, Figural Interpretation Quest; ANA, Anagram Task; STEM, Stem Completion Task. Spearman’s *rho* in square brackets. ***p < .001,**p < .01,*p < .05, ^*p* < .10

**Table 6.** Across-task correlations for proportion shift-count on the 2-item tasks (Pilot Data).

task	AUT	FIQ	ANA	STEM
AUT	—			
FIQ	.37** [.39**]	—		
ANA	.34* [.42**]	.31* [.35*]	—	
STEM	.25^ [.36*]	.55*** [.56***]	.54*** [.48***]	—

AUT, Alternative Uses Task; FIQ, Figural Interpretation Quest; ANA, Anagram Task; STEM, Stem Completion Task. Spearman’s *rho* in square brackets. ***p < .001,**p < .01,*p < .05, ^*p* < .10

To address the second key research question of whether participants showed heightened flexibility for divergent-thinking relative to convergent-thinking tasks, we contrasted the *proportion shift-count* for the divergent versus convergent tasks. There was a substantially higher proportion of shifts for divergent than for convergent tasks. As can be seen from table 3, the means were: AUT proportion shift-count = .67, FIQ proportion shift-count = .71; Anagram proportion shift-count = .52, Stem Completion proportion shift-count = .41.

Table 7 presents repeated-measures analyses of variance (ANOVAs) contrasting the proportion shift-count for each of the divergent tasks (AUT and FIQ) against each of the convergent tasks (ANA and STEM). All four of the pairwise comparisons for proportion shift-count (AUT > ANA, AUT > STEM, FIQ>ANA, FIQ > STEM) were significant (all *p* < .001). The average proportion shift-count for the divergent tasks was .69 compared with an average proportion shift-count of .47 for the convergent tasks, *F*(1, 47) = 69.46, *p* < .001, partial eta^2^ = .60.

**Table 7.** ANOVA results for proportion shift-count comparison of divergent versus convergent tasks (Pilot Data).

comparison and direction of means	*F* ratio	effect size^a^	significance
AUT versus ANA (AUT>ANA)	20.33	.302	*p* < .001
AUT versus STEM (AUT>STEM)	38.63	.451	*p* < .001
FIQ versus ANA (FIQ>ANA)	34.35	.422	*p* < .001
FIQ versus STEM (FIQ>STEM)	82.66	.638	*p* < .001

AUT, Alternative Uses Task; FIQ, Figural Interpretation Quest; ANA, Anagram Task; STEM, Stem Completion Task. Each row presents the results of a repeated measures ANOVA, contrasting the mean proportion shift-count for the two designated tasks.

^a^ Partial eta^2^

### 2.3. Interim conclusion and cognitive task analysis in relation to originality

In the following pre-registered experiment, we again examine if the proportion of shifting is greater for divergent than for convergent thinking tasks. Additionally, we also examine the effects of proportion shift-count on the *quality* of the divergent thought responses that participants generate, asking whether a higher proportion of shifting during the divergent thinking tasks is associated with higher levels of fluency and originality. This is an important applied and theoretical question given suggestions that not just taking a break or a rest but also switching to a new context—here instantiated by a new item within the same overarching task and task rules—may counteract cognitive fixation, and so be a time-efficient way to bolster creative ideation [42,50,51].

The provision of a second item on a divergent thinking task under free-to-choose conditions provides individuals with the opportunity (but *not* the *requirement*) to choose to transition to a different item, or to transition back to the item they were earlier thinking about. The 2-item format thus may offer bottom-up stimulation or support to counter possible fixation on earlier generated ideas [42,52], or a form of indirect incubational ‘time away’ for the emergence of ideas relating to the (not currently attended) item [41,42]. Yet, in our prior research [5], both shift-count and dwell-length were positively associated with originality of responses on the 2-item AUT—suggesting that both shifting one’s generational efforts to a different item, *and* sustaining one’s generational attention on the current item, can contribute to idea originality. Although researchers have emphasized the especial ideational benefits of switching [41], it is also the case that sustained attention on a given item may bring different ideational dividends (see table 1 of Wu & Koutstaal [28] for a detailed cognitive task analysis). For example, remaining longer with a given item can allow executive processes to effectively reach past initially retrieved, highly accessible but less novel or original ideas [14,53,54] and continued persistence in thinking about a given item may set the stage for imaginative/perceptual-motor simulations that require time and effort and that tend to emerge later during participants' think-aloud protocols [14,55]. This suggests that original ideation on the 2-item divergent thinking tasks will depend on the extent to which participants can negotiate these varied pros and cons, with originality bolstered *both* by a higher proportion of shifts *and* by a comparatively longer average dwell-length.

## 3. The current study

In the current pre-registered study, we use the process-based measures of flexibility and persistence offered by the SGT measures of shift-count and dwell-length to further examine both interindividual (trait-like) factors, such as a general predisposition to show a high level of flexibility, and intraindividual (state-like) factors, such as showing varying degrees of persistence across different problem-solving contexts. We use the same four tasks as employed in the preliminary work reported above, that is, two divergent thinking tasks (AUT and FIQ) and two convergent thinking tasks (Anagram, Stem Completion). We also sought to extend our prior findings by including one additional task (an exemplar generation task based on sensory impression information, similar to that previously used by Smith *et al*. [42]) and, as in our earlier study [28], we also assessed participants' meta-cognitive responses to the 2-item versus 1-item tasks. Additionally, in an exploratory spirit, we also seek to further characterize possible patterns of individual differences in SGTs by including both additional cognitive-behavioural assessments of fluid reasoning and vocabulary, and questionnaire-based measures of personality (e.g. openness to experience), prior creativity-related training and activities, and creativity-related beliefs/attitudes (e.g. creative efficacy).

This study was embedded in a larger, separately pre-registered project, the ‘College Wellbeing and Thinking Study’. The key cognitive-behavioural tasks involving assessment of the SGTs and cognitive-behavioural assessments of fluid reasoning and vocabulary took place in a Zoom-based researcher-led session of approximately 90 min. Prior to completing the Zoom-based session, participants independently completed online questionnaires that provided demographic information, and questionnaire-based measures of personality (e.g. openness to experience), prior creativity-related training and activities, and creativity-related beliefs/attitudes (e.g. creativity mindset). Participants included both college students who were 18 years of age or older and early college students (students who were currently enrolled in university courses who were not yet 18 years of age). This allowed exploratory examination of the question of whether SGTs differ for relatively younger participants versus slightly older participants with differing educational pathways, and further assessment of the generalizability of the patterns observed. Although our preliminary SGT study (described above) demonstrated significant across-task correlations with a sample size of 49 participants, to ensure sufficient power and to maximize generalizability, in accordance with our preregistered data collection plan, we continued testing until there were 120 participants with complete SGT data.

### 3.1. Research questions and predictions

There were three primary research questions.

#### 3.1.1. Research Question 1

Do individuals show across-task consistency in how often they choose to shift versus choose to dwell for divergent thinking tasks, convergent thinking tasks and/or both divergent and convergent tasks? *Prediction:* replicating the findings from our pilot study (described above, in §2.2), we predict that individuals will show *across-task consistency* in how often they choose to shift versus choose to dwell for divergent tasks, convergent tasks and both divergent and convergent tasks. Specifically, we expect to observe moderate positive pairwise correlations of the measures of shift-count across the SGT tasks, and moderate positive pairwise correlations of the measures of dwell-length across the SGT tasks; we also expect that most of these pairwise correlations will be separately statistically significant.

#### 3.1.2. Research Question 2

Do individuals autonomously choose to shift more frequently for divergent thinking tasks than for convergent thinking tasks? *Prediction:* replicating the findings from our pilot study (described above, in §2.2), we expect that, for the measure of *proportion shift-count*, individuals will autonomously choose to shift more frequently for the divergent thinking tasks than for the convergent thinking tasks.

#### 3.1.3. Research Question 3

For divergent thinking tasks, does (i) a higher proportion of shifts and (ii) a longer average dwell-length lead to higher ideational fluency (overall number of responses) and/or to more original responses? (That is, how do shift-count versus dwell-length relate to within-task creative performance?). *Prediction:* based on the findings from our 2020 study [5] and the cognitive task analyses (outlined above in §2.3), we expect that, on the divergent thinking tasks, both (i) the proportion shift-count and (ii) the average dwell-length will be significantly positively correlated with ideational fluency and with originality.

## 4. Methods

As noted, this study was preregistered (https://osf.io/gzh86). In accordance with our preregistered data collection plan, we continued testing until there were 120 participants with complete SGT data. Experimental procedures, data preprocessing and data analyses for the three research questions were performed as detailed in the preregistration.

### 4.1. Participants

A total of 128 individuals (102 female, 22 male, 4 other), including 95 regular college students (*M* age 19.85 years, s.d. = 2.41) and 33 early college students (*M* age 16.79 years, s.d. = 0.65) took part. Participants identified as European American or White (60.2%), Asian American or Asian (26.6%), African American or Black (5.5%), Middle Eastern or Arabic (3.9%), Hispanic or Latino/a/x (2.3%), Native American or Alaska (0.8%), and Native Hawaiian or Other (0.8%). Approximately one-third (35.2%) were psychology majors. All participants self-reported as having normal (or corrected) vision and hearing.

The study was conducted in accordance with an approved IRB (Institutional Review Board) protocol of the University of Minnesota (Study 00018504). All participants aged 18 and older gave written informed consent; following written consent of one parent or legal guardian, all participants younger than 18 provided written assent.

### 4.2. Research design and stimuli

The research design was a 2 (task type: divergent or convergent) × 2 (task format: 1-item or 2-item) × 2 (task order: divergent first or convergent first) mixed-factor design, with task type and task format manipulated within subjects and task order between subjects. Task type was manipulated in ‘mini-blocks,’ with two successively presented divergent tasks (Alternative Uses Task, and Figural Interpretation Quest) and two successively presented convergent tasks (Anagram, and Word Stem Completion); the exploratory exemplar generation task based on sensory impression information (Sensory Generation Task) was always the last administered SGT task, and was directly followed by the Meta-Cognitive Questionnaire for all of the prior SGT tasks.

See figure 2 for the stimuli and instructions for each of the five SGT tasks.

### 4.3. Procedure

**Figure 3.**
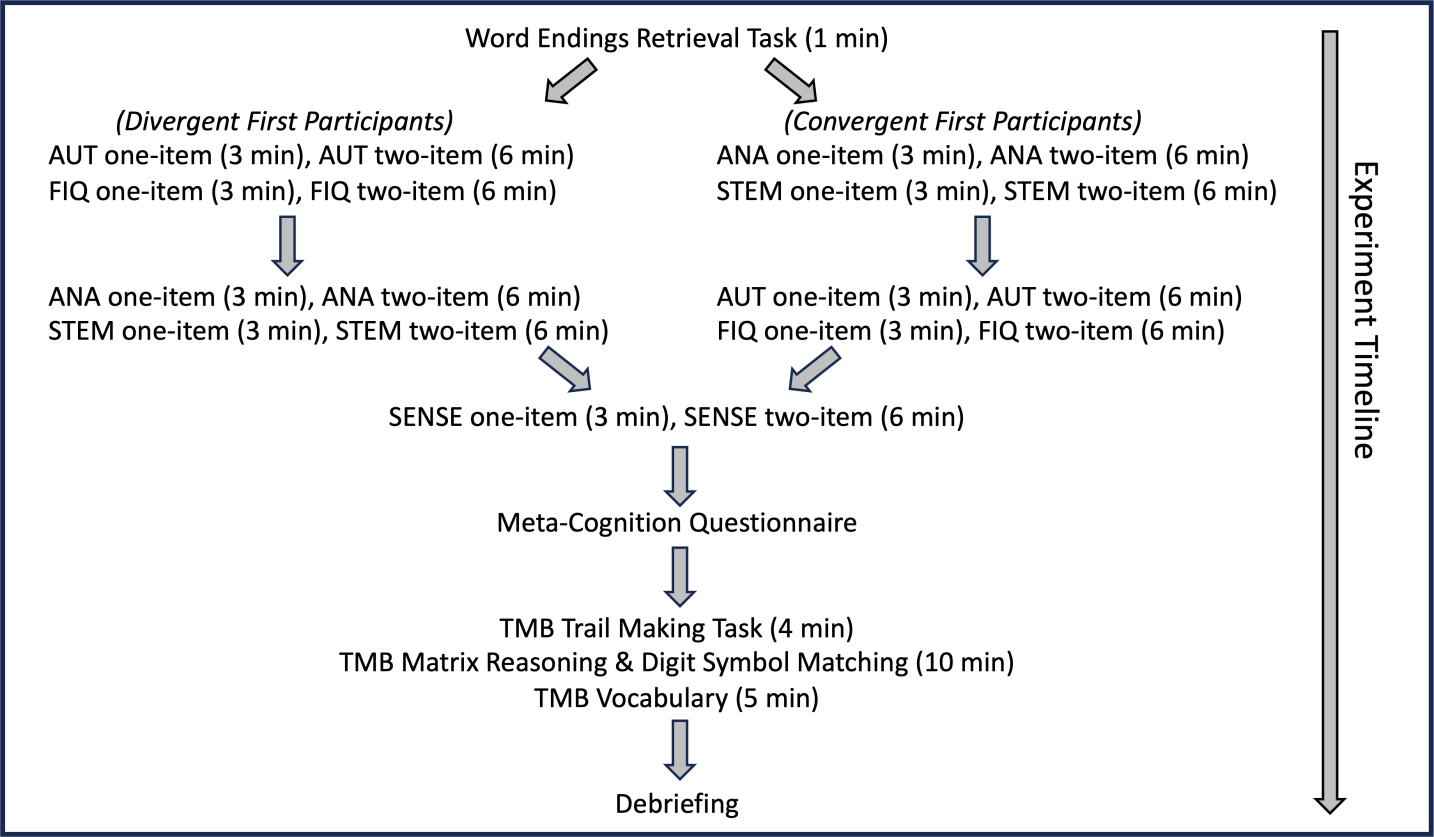
Overview of the experimental procedure. AUT, Alternative Uses Task; FIQ, Figural Interpretation Quest; ANA, Anagram Task; STEM, Stem Completion Task; SENSE, Sensory Generation Task; TMB, Test My Brain.

All participants were tested individually, in a researcher-led Zoom-based session of approximately 90 min. Figure 3 provides an overview of the experimental procedure.

To provide a measure that was equated across conditions, and to help pre-familiarize participants with the study procedures, a Word Endings Retrieval Task was always administered as the first assessment. This measure asked participants to generate as many English words as possible that end in the letters ‘TION’ (task duration of 60 s, as in [56]). For the Self-Guided Transition tasks, the Divergent Tasks were the Alternative Uses Task (AUT) and Figural Interpretation Quest (FIQ); the Convergent Tasks were Anagram (ANA) and Stem Completion (STEM). Each task was first given in the 1-item format, and then 2-item format (procedure and stimuli as in [28]). Task type was varied in mini-blocks such that the divergent tasks were always given in the order of AUT, then FIQ, and convergent tasks were always given in the order of Anagram, then Stem. The sensory generation (SENSE) task, based on Smith *et al*. [42], was always the last administered SGT task, after the divergent and convergent tasks. In the SENSE exemplar-generation task, participants were asked to list things that might lead to specific sensory impressions. The SENSE task also was first given in 1-item format (with the prompt ‘*round*’), and then in 2-item format (with the prompts ‘*cold*’ and ‘*heavy*’).

In accordance with the approved IRB protocol, participants' real-time typed responses to each of these tasks were continuously screen-recorded for later scoring of their shift-count and dwell-length responses. Screen-recording did not include an image of the participant and was discontinued after the SENSE task was completed.

Next, a Meta-Cognitive Questionnaire regarding the SGT tasks (as in [28]) was administered. This questionnaire included nine meta-cognitive questions for each of the five 2-item tasks and was given directly after all of the SGT tasks were completed; the questions per task were given in the same order as the tasks themselves. Some of the meta-cognitive questions asked participants to estimate the *frequency* with which they engaged in specific actions during the 2-item tasks (e.g. how often they switched when they wanted to work on something different or new); other meta-cognitive questions asked participants to rate their *subjective experience* during the 1-item versus 2-item tasks (e.g. the extent to which they found switching between the two items was helpful, and the extent to which they found switching between items was interruptive). (See *Meta-Cognition Questionnaire Responses* for description of the full set of meta-cognitive questions.)

At the end of each session, participants completed three standardized assessments from the Test My Brain (TMB) Open Research and Education online tools [57,58]: TMB Trail Making (4 min), TMB Matrix Reasoning & Digit Symbol Matching (10 min) and TMB Vocabulary (5 min). Also, in the 1–2 weeks prior to the Zoom-based session, participants independently completed several online self-report measures relating to creative ideation, including openness to experience [59,60], beliefs about creativity such as creative self-efficacy [61], and the number and variety of their prior creativity-related training and activities [35,62]. Results for the Test My Brain assessments, and correlations between the prior self-reported survey measures and SGT fluency and process measures are reported in electronic supplementary material, tables S2–S8.

## 5. Results

Data for this study are openly available on Harvard DataVerse. Findings are reported in the following order: (i) descriptive statistics for the Self-Guided Transition Tasks, including overall fluency (correct responses) for the 1-item and 2-item tasks and for the SGT process measures of shift-count, dwell-length and proportion shift-count for the 2-item tasks, (ii) results in relation to each of the three primary research questions and (iii) findings from the Meta-Cognition Questionnaire regarding the SGT tasks. Given that there were no age-group differences of the early versus regular college students on either the SGT tasks or the Test My Brain assessments (see electronic supplementary material, tables S9 and S10), all analyses are reported combining across the full sample.

### 5.1. Descriptive statistics for the self-guided transition tasks

Table 8 gives descriptive statistics (including the mean, 95% confidence interval around the mean, median, standard deviation and *N*) for overall fluency (that is, correct responses) on the five 1-item and five 2-item tasks, including the two divergent thinking tasks (AUT and FIQ), the two convergent thinking tasks (ANA and STEM) and the (exploratory) SENSE task. Table 9 gives the corresponding descriptive statistics for shift-count and dwell-length for the five 2-item tasks and table 10 gives the corresponding descriptive statistics for proportion shift-count. (See figure 1 for a schematic depiction of how shift-count and dwell-length were calculated, and electronic supplementary material, table S12 for additional SGT process scoring explanation.)

**Table 8.** Descriptive statistics for fluency on all the 1-item and 2-item tasks.

task	mean	lower 95% CI	upper 95% CI	median	std. dev.	*N*
*one item*						
AUT	7.10	6.53	7.67	7.00	3.24	128
FIQ	8.03	7.43	8.64	7.00	3.46	128
ANA	9.80	8.95	10.65	9.00	4.85	128
STEM	10.07	9.29	10.85	9.00	4.46	128
SENSE	15.39	14.13	16.65	15.00	7.22	128
*two item^a^*						
AUT	16.48	15.28	17.69	15.00	6.89	128
FIQ	17.02	15.73	18.30	16.00	7.37	128
ANA	16.43	15.02	17.84	16.00	8.06	128
STEM	33.25	31.42	35.08	32.00	10.48	128
SENSE	28.04	25.89	30.18	26.00	12.26	128

AUT, Alternative Uses Task; FIQ, Figural Interpretation Quest; ANA, Anagram Task; STEM, Stem Completion Task; SENSE, Sensory Generation Task.

^a^ Two item values are based on the total fluency for the two items.

**Table 9.** Descriptive statistics for the SGT process measures for the 2-item tasks.

task and measure	mean	lower 95% CI	upper 95% CI	median	std. dev.	*N*	*N* outliers (IDs^a^)
AUT shift-count	5.44	4.91	5.97	5.00	3.01	125	—
AUT dwell-length	3.03	2.66	3.41	2.50	2.14	125	6 (230, 272, 274, 265, 276, 235)
FIQ shift-count	6.66	6.08	7.25	7.00	3.28	125	1 (233)
FIQ dwell-length	2.49	2.19	2.80	2.11	1.71	125	5 (230, 294, 329, 290, 243)
ANA shift-count	3.53	3.14	3.93	3.00	2.21	124	—
ANA dwell-length	4.26	3.83	4.69	3.69	2.44	124	2 (253, 225)
STEM shift-count	5.60	4.63	6.56	4.00	5.42	124	3 (295, 216, 309)
STEM dwell-length	7.58	6.67	8.49	6.09	5.12	124	—
SENSE shift-count	5.60	5.07	6.14	5.00	3.03	124	—
SENSE dwell-length	5.25	4.48	6.02	3.73	4.31	124	5 (294, 269, 230, 274, 223)

AUT, Alternative Uses Task; FIQ, Figural Interpretation Quest; ANA, Anagram Task; STEM, Stem Completion Task; SENSE, Sensory Generation Task.

^a^ IDs, participant identification numbers for outlier values; all outlier values were higher than the mean for the designated measure. Note that outlier values are not errors—and, indeed, individual differences or variation in the measures of dwell-length and shift-count are of primary research interest. Thus, the descriptive statistics are calculated including all participants, but the median is also provided; additionally, all subsequent correlational analyses report both Pearson’s correlation and Spearman’s nonparametric rank correlation coefficient (*rho),* which is less influenced by outlier values.

**Table 10.** Descriptive statistics for the proportion shift-count SGT measures for the 2-item tasks.

task	mean	lower 95% CI	upper 95% CI	median	std. dev.	*N*	*N* outliers (IDs^a^)
AUT proportion shift	.36	.32	.39	.33	.19	125	—
FIQ proportion shift	.41	.38	.44	.40	.17	125	—
ANA proportion shift	.23	.21	.26	.20	.15	123^b^	1 (290)
STEM proportion shift	.18	.15	.21	.13	.16	124	1 (295)
SENSE proportion shift	.24	.21	.26	.22	.15	124	—

AUT, Alternative Uses Task; FIQ, Figural Interpretation Quest; ANA, Anagram Task; STEM, Stem Completion Task; SENSE, Sensory Generation Task.

^a^ IDs, participant identification numbers for outlier values; all outlier values were higher than the mean for the given measure.

^b^ One participant excluded with a 2-item Anagram score of zero.

### 5.2. Findings for Research Question 1

Do individuals show across-task consistency (trait-like flexibility or persistence) in how often they choose to shift versus choose to dwell for divergent thinking tasks, convergent thinking tasks and/or both divergent and convergent tasks?

The across-task correlations in shift-count, dwell-length and proportion shift-count are provided in tables 11, 12 and 13, respectively.

**Table 11.** Across-task correlations for shift-count on the 2-item tasks.

task	AUT	FIQ	ANA	STEM	SENSE
AUT	—				
FIQ	.46^***^ [.43^***^]	—			
ANA	.01 [.06]	.19* [.21*]	—		
STEM	.39^***^ [.41^***^]	.25** [.25**]	.16 [.28**]	—	
SENSE	.35^***^ [.33^***^]	.30^***^ [.27**]	.13 [.17]	.45^***^ [.46^***^]	—

AUT, Alternative Uses Task; FIQ, Figural Interpretation Quest; ANA, Anagram Task; STEM, Stem Completion Task; SENSE, Sensory Generation Task. Spearman’s *rho* in square brackets. ***p < .001, **p < .01, *p < .05

**Table 12.** Across-task correlations for dwell-length on the 2-item tasks.

task	AUT	FIQ	ANA	STEM	SENSE
AUT	—				
FIQ	.59^***^ [.36^***^]	—			
ANA	.08 [.05]	.17 [.18*]	—		
STEM	.40^***^ [.34^***^]	.31^***^ [.37^***^]	.25** [.27**]	—	
SENSE	.55^***^ [.45^***^]	.56^***^ [.36^***^]	.19* [.19*]	.47^***^ [.43^***^]	—

AUT, Alternative Uses Task; FIQ, Figural Interpretation Quest; ANA, Anagram Task; STEM, Stem Completion Task; SENSE, Sensory Generation Task. Spearman’s *rho* in square brackets. ***p < .001, **p < .01, *p < .05

**Table 13.** Across-task correlations for proportion shift-count on the 2-item tasks.

task	AUT	FIQ	ANA	STEM	SENSE
AUT	—				
FIQ	.36^***^ [.38^***^]	—			
ANA	.02 [.02]	.15 [.17]	—		
STEM	.34^***^ [.37^***^]	.31^***^ [.34^***^]	.12 [.26**]	—	
SENSE	.34^***^ [.37^***^]	.39^***^ [.34^***^]	.07 [.14]	.43^***^ [.45^***^]	—

AUT, Alternative Uses Task; FIQ, Figural Interpretation Quest; ANA, Anagram Task; STEM, Stem Completion Task; SENSE, Sensory Generation Task. Spearman’s *rho* in square brackets. ***p < .001, **p < .01, *p < .05

From tables 11, 12 and 13, it can be seen that, for all three SGT process measures—shift-count, dwell-length and proportion shift-count—there are significant across-task correlations for all of the pairwise relations of AUT, FIQ, STEM and SENSE, including across the two divergent tasks (AUT, FIQ), the convergent but relatively open-ended STEM task, and the exploratory SENSE task. For shift-count, the six pairwise correlations for these four tasks (AUT, FIQ, STEM and SENSE) range between *r* = .31 and *r* = .59, with an average Fisher *Zr* = .39 (for the three pairwise correlations of shift-count when excluding the exploratory SENSE task, average Fisher *Zr* = .39). For dwell-length, the corresponding six pairwise correlations range between *r* = .31 and *r* = .43, with an average Fisher *Zr* = .53 (excluding SENSE, average Fisher *Zr* = .47). For proportion shift-count, these six pairwise correlations range between *r* = .31 and *r* = .43, with an average Fisher *Zr* = .38 (excluding SENSE, average Fisher *Zr* = .35).

By contrast, also from tables 11–13, it can be seen that the SGT process measures for the most highly constrained convergent task, ANA, only weakly and in most cases nonsignificantly correlates with SGT process measures of the four other tasks, with the strongest correlation being that between ANA dwell-length with STEM dwell-length—that is, the other primarily verbal and letter-based task. Across the four pairwise correlations of ANA with each of the remaining tasks, for shift-count, the average Fisher *Zr* = .12; for dwell-length, the average Fisher *Zr* = .17, and for proportion shift-count, the average Fisher *Zr* = .09. Excluding the correlations with SENSE, these averaged Fisher *Zr* correlations = .12, .17 and .10, respectively.

Summarizing the findings in relation to Research Question 1: individuals *do* show strong and significant across-task (‘trait-like’) consistency in how often they choose to shift versus choose to dwell for divergent tasks (AUT, FIQ), and also for an open-ended (not highly constrained) convergent task (STEM) as well as for the SENSE task. By contrast, for the most highly constrained convergent task (ANA), across-task consistency in how often individuals choose to shift versus choose to dwell with the other (more open-ended and not tightly constrained tasks) is slight to modest. This suggests that while there are intraindividual (trait-like) factors that lead participants to show similar degrees of shifting versus dwelling across various tasks, these intraindividual tendencies are *also* modified by the specific demands of the task they are given, with the effects of such task-based or context-based demands particularly apparent for tasks that are highly constrained.

### 5.3. Findings for Research Question 2

Do individuals autonomously choose to shift more frequently for divergent thinking tasks than for convergent thinking tasks?

Table 14 summarizes the statistical comparisons (repeated measures ANOVAs), separately contrasting the *mean proportion shift-count* for each of the six different pairs of tasks, including the divergent thinking tasks (AUT, FIQ) versus the convergent thinking tasks (ANA, STEM), and also the exploratory SENSE generation task.

**Table 14.** ANOVA results for proportion shift-count comparisons of divergent versus convergent tasks.

comparison and direction of means	*F* ratio	effect size^a^	significance
AUT versus ANA (AUT>ANA)	*F*(1, 122) = 31.97	.208	*p* < .001
AUT versus STEM (AUT>STEM)	*F*(1, 123) = 96.14	.439	*p* < .001
FIQ versus ANA (FIQ>ANA)	*F*(1, 122) = 89.87	.424	*p* < .001
FIQ versus STEM (FIQ>STEM)	*F*(1, 123) = 178.00	.591	*p* < .001
SENSE versus ANA (SENSE = ANA)	*F*(1, 122) = 0.06	.001	*p* = .80
SENSE versus STEM (SENSE>STEM)	*F*(1, 123) = 15.65	.113	*p* < .001

AUT, Alternative Uses Task; FIQ, Figural Interpretation Quest; ANA, Anagram Task; STEM, Stem Completion Task; SENSE, Sensory Generation Task. Each row presents the results of a repeated measures ANOVA, contrasting the mean proportion shift-count for the two designated tasks.

^a^ Partial eta^2^

As can be seen from table 14, consistent with the pilot study results and in alignment with the preregistered hypotheses, proportion shift-count for each of the divergent thinking measures (AUT and FIQ, proportion shift-count of .36 and .41, respectively) significantly exceeds that for each of the two main convergent thinking tasks (ANA and STEM, proportion shift-count of .23 and .18, respectively), all *p* < .001. This strongly points to the *task adaptivity* of participants' responding, with participants demonstrating an increased proportion of shifting for the more open-ended divergent-search tasks than for the convergent tasks. The exploratory inclusion of the Sensory Generation task suggests a higher proportion of shifting for the SENSE task (proportion shift-count of .24) than for the STEM task, but not for the SENSE relative to the ANA task.

### 5.4. Findings for Research Question 3

For divergent thinking tasks, does (i) a higher proportion shift-count and (ii) a longer average dwell-length lead to higher ideational fluency (overall number of correct responses) and/or to more original responses? (That is, how do shift-count versus dwell-length relate to within-task creative performance?)

Participants' responses to the two main divergent thinking tasks (AUT and FIQ) were collaboratively classified by three raters for response fluency (that is, the number of correct and complete responses). For scoring originality, each response was then further collaboratively classified into one of three categories: typical (that is, highly common and very frequently given responses, awarded 0 points for originality), somewhat nontypical (that is, relatively less common or less frequently provided responses, awarded 1 point for originality), or creative (that is, original and infrequently provided responses, awarded 2 points for originality). A similar classification approach was used for the exploratory ‘SENSE’ task, but with a category of ‘metaphorical’ responses substituted for the ‘creative’ category. An originality score for each task was then obtained by summing points for the nontypical and creative responses. (See electronic supplementary material, table S1, for pairwise correlations between the originality scores for all the divergent thinking tasks, all *p* < .007).

The correlation between each of the three SGT process measures (Shift-Count, Dwell-Length and Proportion Shift-Count) was then found with overall fluency and originality for each divergent-thinking task. Tables 15, 16 and 17 provide these correlations for the AUT, FIQ and SENSE tasks, respectively.

**Table 15.** Correlations between SGT process measures and performance for the AUT.

task	AUT shift	AUT dwell	AUT fluency	AUT originality	AUT prop. shift
AUT shift-count	—				
AUT dwell-length	−.40*** [−.42***]	—			
AUT fluency	.35*** [.41***]	.50*** [.49***]	—		
AUT originality	.29** [.32***]	.41*** [.42***]	.86*** [.85***]	—	
AUT proportion shift-count	.69*** [.66***]	−.63*** [−.87***]	−.34*** [−.34***]	−.30*** [−.32***]	—

AUT, Alternative Uses Task; Spearman’s *rho* in square brackets. ***p < .001, **p < .01, *p < .05

**Table 16.** Correlations between SGT process measures and performance for the FIQ.

task	FIQ shift	FIQ dwell	FIQ fluency	FIQ originality	FIQ prop. shift
FIQ shift-count	—				
FIQ dwell-length	−.37*** [−.45***]	—			
FIQ fluency	.54*** [.56***]	.40*** [.37***]	—		
FIQ originality	.45*** [.47***]	.37*** [.26**]	.81*** [.79***]	—	
FIQ proportion shift-count	.55*** [.61***]	−.64*** [−.92***]	−.30*** [−.24**]	−.22* [−.16]	—

FIQ, Figural Interpretation Quest; Spearman’s *rho* in square brackets. ***p < .001, **p < .01, *p < .05

**Table 17.** Correlations between SGT process measures and performance for the SENSE task.

task	SENSE shift	SENSE dwell	SENSE fluency	SENSE originality	SENSE prop. shift
SENSE shift-count	—				
SENSE dwell-length	−.53*** [−.57***]	—			
SENSE fluency	.05 [.02]	.63*** [.71***]	—		
SENSE originality	−.02 [.03]	.43*** [.43***]	.65*** [.64***]		
SENSE proportion shift-count	.63*** [.69***]	−.69*** [−.96***]	−.60*** [−.64***]	−.38*** [−.37***]	—

SENSE, Sensory Generation Task; Spearman’s *rho* in square brackets. ***p < .001, **p < .01, *p < .05

Focusing first on the results for shift-count and dwell-length in relation to fluency and originality, from table 15 we can see that, as predicted, *both* shift-count and dwell-length are positively and significantly correlated with AUT fluency and also with originality of AUT responses. From table 16, we can see that the same pattern is found for FIQ: both shift-count and dwell-length are significantly positively correlated with FIQ fluency and also with FIQ originality. Next, from table 17, we see that only dwell-length (and not shift-count) is significantly positively correlated with SENSE fluency and SENSE originality. Last, turning our attention to the results for *proportion shift-count*, it can be seen that proportion shift-count is significantly negatively related to fluency for all three tasks, and also negatively related to originality for all three tasks (significantly so for AUT and SENSE).

Collectively, these findings indicate that within-task creative ideation depends at least as much on dwelling as on shifting. Considering the measure of originality of responses specifically, the consistent positive correlations between dwell-length and participants' originality scores for all three tasks (correlations of .41, .37 and .43 for AUT, FIQ and SENSE, respectively) coupled with the negative correlations of proportion shift-count with originality (correlations of -.30, -.22 and -.39, respectively) together counter claims for the predominant benefits of shifting, and align with the proposal that *both flexibility and persistence* may benefit creative thinking.

### 5.5. Meta-Cognition Questionnaire responses

The first four of the questions on the Meta-Cognition Questionnaire asked participants to estimate the *frequency* with which they engaged in specific actions during the 2-item tasks (zero times, 1 time, 2 times, 3 times, or 4 or more times). These meta-cognitive estimated frequency questions were modified to align with each of the different tasks. For instance, for the AUT, the questions were as follows: *Switch Return* = I tried to switch to think about a use for the other different object, but when I couldn't come up with anything, I returned to the original object; *Switch Out of Ideas* = I switched from one object to the other when I could not think of solutions for the current object; *Switch for New* = I switched when I wanted to work on something different or new; *Switch to try Transfer* = When I found a solution for one object, I tried to see if it would apply to the other object.

Table 18 provides descriptive statistics for these meta-cognitive estimated frequency questions for all five tasks (AUT, FIQ, ANA, STEM and SENSE).

**Table 18.** Descriptive statistics for meta-cognitive estimated frequency of responses by task.

task/question	mean	lower 95% CI	upper 95% CI	median	std. dev.	*N*
AUT-switch return	2.96	2.76	3.16	3.00	1.13	126
FIQ-switch return	3.13	2.94	3.33	4.00	1.09	127
ANA-switch return	2.97	2.77	3.17	3.00	1.13	128
STEM-switch return	2.86	2.65	3.07	3.00	1.21	127
SENSE-switch return	2.76	2.55	2.97	3.00	1.20	127
AUT-switch out of ideas	3.47	3.32	3.62	4.00	0.86	126
FIQ-switch out of ideas	3.47	3.33	3.62	4.00	0.82	127
ANA-switch out of ideas	3.52	3.38	3.66	4.00	0.80	128
STEM-switch out of ideas	3.24	3.07	3.41	4.00	0.96	127
SENSE-switch out of ideas	3.31	3.16	3.47	4.00	0.90	127
AUT-switch for new	2.44	2.20	2.69	3.00	1.37	126
FIQ-switch for new	2.52	2.28	2.76	3.00	1.38	127
ANA-switch for new	2.61	2.37	2.85	3.00	1.36	128
STEM-switch for new	2.63	2.40	2.86	3.00	1.31	127
SENSE-switch for new	2.40	2.15	2.65	3.00	1.42	127
AUT-switch to try transfer	2.10	1.83	2.38	2.00	1.57	126
FIQ-switch to try transfer	2.28	2.02	2.55	2.00	1.51	127
ANA-switch to try transfer	1.78	1.50	2.07	1.00	1.63	128
STEM-switch to try transfer	1.85	1.56	2.14	2.00	1.67	127
SENSE-switch to try transfer	1.76	1.48	2.03	2.00	1.59	127

AUT, Alternative Uses Task; FIQ, Figural Interpretation Quest; ANA, Anagram Task; STEM, Stem Completion Task; SENSE, Sensory Generation Task. Participants indicated the frequency with which the designated events happened during each task on a scale from zero times, 1 time, 2 times, 3 times, or 4 or more times.

From table 18 we observe that, across all tasks, participants reported frequently switching to the other item when they could not think of solutions to the current item (median reported switch of 4 times for all five tasks). They somewhat less often reported switching when they wanted to work on something different or new (median reported switch of 3 times for all tasks) or reported *re-visiting* an item due to lack of success in generating responses for the other item (median reported switch of 3 times except for FIQ, where the median was 4). Overall, participants less often and infrequently reported trying to see if a solution for one item could apply to the other item (median of 1 or 2 times).

A subset of questions on the Meta-Cognition Questionnaire asked participants to rate their *subjective perceptions* regarding the 1-item versus 2-item tasks. Ratings to these questions were made on a 5-point scale: 1 = Strongly disagree; 2 = Disagree; 3 = Neither agree nor disagree; 4 = Agree; 5 = Strongly agree. Participants also had the option of responding ‘Not applicable’; not applicable responses are excluded. The phrasing of the questions was modified for each task; for instance, for the AUT, the questions were as follows: *Two Set Challenging* = Compared with the one-object task, I found the two-object task was more challenging; *Two Set Enjoyable* = Compared with the one-object task, I found the two-object task was more enjoyable; *Stay with Easier* = I found one object was easier to generate uses for than the other object, so I stayed with the easier object longer; Switching Helpful = I found switching between the two objects was helpful; *Switching Interruptive* = I found switching between the two objects was interruptive. Table 19 provides the descriptive statistics for the meta-cognitive subjective rating questions separately for the five tasks (AUT, FIQ, ANA, STEM and SENSE).

**Table 19.** Descriptive statistics for meta-cognitive subjective ratings by task.

task/question	mean	lower 95% CI	upper 95% CI	median	std. dev.	*N*
AUT-two set challenging	2.43	2.23	2.63	2.00	1.11	122
FIQ-two set challenging	2.52	2.31	2.73	2.00	1.16	121
ANA-two set challenging	2.48	2.26	2.69	2.00	1.20	121
STEM-two set challenging	2.20	2.01	2.39	2.00	1.06	122
SENSE-two set challenging	2.65	2.44	2.86	2.00	1.19	123
AUT-two set enjoyable	3.99	3.81	4.17	4.00	0.99	122
FIQ-two set enjoyable	3.89	3.70	4.09	4.00	1.09	121
ANA-two set enjoyable	4.10	3.90	4.30	4.00	1.11	121
STEM-two set enjoyable	4.36	4.20	4.52	5.00	0.91	122
SENSE-two set enjoyable	3.80	3.59	4.00	4.00	1.13	123
AUT-stay with easier	3.59	3.40	3.78	4.00	1.04	122
FIQ-stay with easier	3.60	3.42	3.79	4.00	1.02	121
ANA-stay with easier	3.83	3.66	4.01	4.00	0.97	121
STEM-stay with easier	3.71	3.51	3.92	4.00	1.13	122
SENSE-stay with easier	3.88	3.70	4.05	4.00	0.98	123
AUT-switching helpful	4.14	3.98	4.30	4.00	0.89	122
FIQ-switching helpful	3.93	3.74	4.13	4.00	1.07	121
ANA-switching helpful	4.09	3.89	4.29	4.00	1.96	121
STEM-switching helpful	4.31	4.16	4.46	4.00	0.84	122
SENSE-switching helpful	4.00	3.82	4.18	4.00	1.01	123
AUT-switching interruptive	2.20	2.04	2.37	2.00	0.94	122
FIQ-switching interruptive	2.17	1.98	2.37	2.00	1.08	121
ANA-switching interruptive	2.29	2.08	2.50	2.00	1.18	121
STEM-switching interruptive	1.99	1.82	2.17	2.00	0.98	122
SENSE-switching interruptive	2.25	2.07	2.43	2.00	1.01	123

AUT, Alternative Uses Task; FIQ, Figural Interpretation Quest; ANA, Anagram Task; STEM, Stem Completion Task; SENSE, Sensory Generation Task. Ratings were made on a 5-point scale: 1 = Strongly disagree; 2 = Disagree; 3 = Neither agree nor disagree; 4 = Agree; 5 = Strongly agree. Participants also had the option of responding ‘Not applicable’; not applicable responses are excluded.

From table 19 we can see that participants disagreed that, compared with the 1-item task, the 2-item tasks were more challenging (median value of 2 for all five tasks) and agreed or strongly agreed that the 2-item tasks were more enjoyable (median values of 4 or 5 for all tasks). Participants generally agreed that they stayed longer with the easier of the two items (median of 4 for each task). For all five tasks participants agreed that switching was helpful (median value of 4 for each task) and disagreed that switching was interruptive (median value of 2 for each task).

### 5.6. Comparative fluency performance on the 1-item versus 2-item tasks

Were there switch costs associated with the 2-item compared with the 1-item tasks? Given that participants were allotted twice as much time for the 2-item versus the 1-item task, then if there was neither a cost nor a benefit from the 2-item format, mean performance (fluency) on the 2-item task as a proportion of performance on the corresponding 1-item task would be close to 2.0. Calculating these values for the five tasks, fluency for the 2-item tasks numerically exceeded what might have been anticipated based on the 1-item task performance for three of the tasks, including the AUT (estimate of 2.32), the FIQ (estimate of 2.12) and STEM (estimate of 3.30), but not ANA (estimate of 1.68) or SENSE (estimate of 1.82). Possible implications of these exploratory (and exclusively descriptive) comparisons of the number of correct responses (fluency) during the 1-item versus 2-item task format, as well as some important caveats, are taken up in §6.

## 6. Discussion

The current study aimed to answer the open question, raised by earlier research (reviewed in §1), regarding the extent to which individuals show a trait-like predisposition towards flexibility or towards persistence across different task contexts, including tasks that vary in level of constraint, and that call upon predominantly convergent thinking versus predominantly divergent thinking. To examine how different types of stimuli and constraints may impact individuals' predisposition towards flexibility or towards persistence we examined five Self-Guided Transition tasks—each with our process-based measures of flexibility (shift-count) and persistence (dwell-length). Two of the tasks primarily called on divergent thinking processes, including the conceptually prompted Alternative Uses Task (AUT), and the perceptually prompted Figural Interpretation Quest (FIQ); two tasks predominantly called on convergent thinking, including the quite tightly constrained Anagram (ANA) problem-solving task and a relatively loosely constrained Word Stem Completion (STEM) task; the fifth (exploratory) task was the comparatively open-ended Sensory Generation Task (SENSE). Crucially, while introducing this heterogeneity in the nature of the tasks to assess how generalizable the findings were across comparatively unconstrained versus constrained task contexts, unlike in the earlier studies of Mekern *et al*. [10] or von Helversen [11], we held constant numerous other aspects of the tasks, including the task duration, stimulus display format, response input method, and—most notably—the operationalization of the measures of flexibility (shift-count) and persistence (dwell-length). We here report four central findings.

First, with one exception (discussed later), individuals demonstrated strong and significant *across-task consistency* in how often they chose to shift versus chose to dwell for both divergent- and convergent-thinking tasks. This provides strong support for an interindividual (trait-like) predisposition towards flexibility versus persistence. The clear across-task consistency in participants' shifting versus dwelling that we observed—across varied conceptual, verbal and visual stimuli—is especially notable in that the assessment of shifting versus dwelling was based on each participant’s own, autonomous, in-the-moment choices to continue directing their problem-solving efforts towards a given item or, instead, to transition to devoting their thought and attention to the alternative item. The shift-count and dwell-length measures circumvent the need to make retrospective inferences about the participants' ideational search that are based on researcher-determined categorizations or ‘clustering’ rules, such as whether two successive responses belong to the same semantic category [13], are the same shape or specific use [14], or reflect the same visuo-motor strategy such as figural rotation [15,16]. Shift-count and dwell-length thus provide a unique and individualized assessment of each participant’s overall propensity towards flexibility versus persistence during their problem-solving performance.

Second, despite such across-task consistency, there was also significant *task- or context-related modulation* of flexibility. As revealed in the proportion shift-count measure, participants autonomously chose to shift significantly more frequently for the comparatively open-ended divergent thinking tasks (AUT and FIQ) than on the convergent thinking tasks (ANA and STEM). Such context-based modulation of flexibility replicates the findings observed in our preliminary data (reported in §2) and is highly consistent with the more recent neurocognitive integrative review of Zhang *et al*. [63], proposing that whereas divergent thinking (and also insight solutions in convergent thinking) may rely on, and benefit from, a comparatively relaxed cognitive control setting that promotes cognitive flexibility, convergent analytical thinking may, instead, depend on, and benefit from, a tighter cognitive control setting that enables persistence. The current demonstration that participants autonomously chose to shift significantly more frequently for the comparatively open-ended, divergent thinking tasks than on the convergent thinking tasks—combined with the further observation that individuals also showed strong and significant *across-task consistency* in how often they chose to shift versus chose to dwell—provides direct empirical support for the claim of Mekern and colleagues [64, p. 52] that ‘some people tend to be more persistent where others tend to be more flexible, but the same person may also sometimes tend to be more persistent and sometimes more flexible, depending on situational demands.’

Third, performance on the divergent tasks—as assessed by both the number of ideas generated (fluency) and the originality of those ideas—was positively correlated with both shifting and dwelling. That is, *both* shift-count (switching one’s attention and ideational search efforts away from the current item to the second of two concurrently available prompts) *and* dwell-length (persevering in working on the current item, continuing to direct one’s focus, cognitive effort, and ideational search towards the current task item) contributed to the number and originality of ideas generated on the divergent thinking tasks. This finding closely coheres with several earlier (and newly emerging) theoretical accounts of creative problem-solving that emphasize the contributions of both flexibility and persistence to the process of generating novel yet useful and valuable ideas [2–8,65,66]. Whereas shifting may help to overcome or minimize cognitive fixation [40–42], and can provide external cues that stimulate thinking in a new direction [52], overly frequent shifting may lead to output interference (good ideas may get lost before they can be output), interfere with deeper search and concentrated attentional effort [53], or preclude extended perceptual–conceptual simulation [55]. Thus originality may be best promoted via the dynamically attuned and cognitively controlled incorporation of both shifting and dwelling.

Fourth, participants' retrospective meta-cognitive reports suggested that, contrary to what might be expected based on the well-known switch costs incurred during externally cued task-switching paradigms [67,68] and also observed during voluntary task-switching paradigms [69], across all five (quite varied) *item-switching* tasks, participants viewed the 2-item format that allowed autonomously chosen shifting to be more enjoyable than the 1-item format. They also perceived the option of transitioning to the second provided task-item as helpful to their ideational performance.

On the one hand, participants' retrospective meta-cognitive reports, revealing a preference for the 2-item over the 1-item format, may partially reflect a differentiation between ‘cognitive work’ and t*he subjective experience* that accompanies such work [70]. For example, although there may have been switching costs associated with the 2-item format, these costs may have been attentionally outweighed by countervailing benefits offered by the 2-item format [28], such as alternative bottom-up information stimulating participants' idea generation [38,41,52] or altering their idea search set [42]. On the other hand, comparisons of participants' performance on the 1-item versus 2-item tasks (§5.6) revealed that fluency for the 2-item tasks numerically exceeded what might have been anticipated based on the 1-item task performance for three of the tasks, including the AUT, the FIQ and STEM, but not ANA or SENSE.

These findings—suggesting that participants' meta-cognitive preferences were also often accompanied by actual performance benefits—point to the possible methodological and pedagogical value of structuring especially divergent thinking or less-tightly constrained tasks in the 2-item compared with a 1-item format. For instance, working on the second of two items during the 2-item format may provide a helpful interlude for ‘forgetting fixation’ on the first item [50], or more generally some time away from directly working on the first item may enable the incipient emergence or incubation of fresh ideas [42,71]. More broadly, these findings underscore the potential value of offering participants the *autonomy* to choose to address their attention and efforts during idea search towards an alternative within the same task or general content domain [42,50]. Indeed, experiments on task choice have shown that self-determined (free) rather than imposed (forced) choices may lead to improved task performance and more effective shielding from incidental environmental variation [72]. And providing the opportunity for a heightened sense of autonomy in complex tasks such as reading comprehension has been found to lead to lower feelings of mental effort, lower mental fatigue and higher positive valence [73]. Nonetheless, empirical replication and caution in interpretation of the current comparison of participants' fluency performance on the 1-tem versus 2-item tasks (reported in §5.6) is necessary for two reasons. First, given the current study’s main goals, to ensure that participants were sufficiently familiar with each task before obtaining SGT measures for the task, the 2-item format always followed the 1-item format. Thus, to the extent that there were general practice-related improvements on a task, these would favour performance on the 2-item version (although note, too, that fatigue effects might work against such an effect). Second, the 2-item stimuli within each task were held constant across all participants (rather than counterbalanced across the formats within the task) so performance differences may partially reflect item-related differences.

Based on the meta-cognitive frequency estimates, participants reported that they sometimes—but relatively rarely— chose to switch to the other item in order to see if they might transfer ideas to the other item. The question of whether participants use shifting during the 2-item AUT to access alternative ideas has recently been explored by Yang *et al*. [74]. These researchers noted that the process of drawing inspiration from a previous items’s uses while shifting between items might involve both conscious and unconscious attentional processes, with conscious shifts likely to draw upon executive control and working memory abilities and unconscious shifting potentially arising from more spontaneous mind-wandering. Yang *et al*. [74] reported that participants who were (on separate tasks) independently characterized as both high in working memory capacity and showing high levels of mind wandering showed a significant positive correlation between shifting and (separately coded) inter-item references on the AUT. This pattern was not found for participants with other latent profiles of low working memory and low mind-wandering, or high working memory and low mind-wandering, and the different profile groups did not show significant differences in the simple measure of shift-count. Participants with this profile (high in working memory capacity and high in mind wandering) also demonstrated significant correlations of within-item dwell-length and AUT performance, again pointing to the benefits of persistence in creative ideation.

Although there was clear within-participant consistency in shifting and dwelling across four of the five SGT tasks, the Anagram task was a notable exception. Whereas for shift-count, dwell-length and proportion shift-count the six pairwise correlations for the four tasks of AUT, FIQ, STEM and SENSE were all statistically significant, with across-task averaged (Fisher *Zr*) correlations of .39, .53 and .38 respectively, the correlations of these four tasks with Anagram, although also numerically positive, were markedly weaker, with averaged (Fisher *Zr*) correlations of .12, .17 and .09, respectively. From a cognitive-task analysis perspective, the Anagram task differs from the other tasks in the number and nature of the constraints it places on idea search, including especially intensive demands placed on working memory (cf. [75]). Specifically, in the current study, the Anagram task involved nine possible letters for each item, subsets of which could be rearranged into numerous different possible solution configurations. This contrasts with the other SGT tasks, where each item was indicated by a word denoting a common object (AUT), an ambiguous visual shape (FIQ) or two letters for the beginning of each word (STEM). Thus the Anagram task—as constituting an especially stringent problem-solving context with unique cognitive control demands—might be especially well-suited to demonstrate not between individual differences in flexibility versus persistence, but rather more *uniformly* manifested ‘state-like’ effects.

This may cohere well with the conclusions that Payne *et al*. [76] reached, based on an early series of studies using Anagram problem-solving with a 2-item task structure, and where, as in the SGT paradigm, participants were free to move back-and-forth between the two provided letter sets. Payne *et al*. [76] concluded that their most fundamental observation was that ‘when participants were free to allocate their time as they wished across a pair of tasks, most chose to switch between the tasks rather frequently’ [76, p. 385], and further suggested that they may have uncovered ‘some surprising findings concerning a rather universal behavioural tendency, that is, discretionary interleaving of independent tasks’ [76, p. 386]. Indeed, of all five tasks used in the current work, the Anagram task showed the weakest across-task correlations in shift-count, dwell-length and proportion shift-count, suggesting that between-item transitioning was the least influenced by individual differences (trait predisposition towards flexibility/persistence) and the most strongly determined by the task context itself.

Although the current research also included the Word Stem task as an additional convergent-thinking task, relative to the Anagram task, the STEM task was still fairly open-ended, with potential (4-letter English word) solutions constrained by only the initial two letters given in the stem (e.g. *AL–*). Future research might adopt additional more tightly constrained tasks (e.g. placing additional limits on permitted responses for the STEM task to further narrow the search set) to assess if strong contextually modulated effects on flexibility versus persistence generalize to tasks beyond Anagram. It might also be valuable to vary the stimulus-related demands of the Anagram task itself (e.g. by reducing the number of possible letters per item), or to present the permissible letters that are to be recombined themselves as words [77], thereby making each of the items more unified and thus as readily ‘held in mind’ as the stimuli for the other tasks.

The findings for the exploratory Sensory Generation Task, indicating no fluency benefit from the 2-item compared with the 1-item format, and perhaps some detriment to fluency, may appear inconsistent with earlier findings reported by Smith *et al*. [42]. These earlier researchers suggested that shifting benefits should be especially clear for conceptual/semantic categories that are less stable or that are more flexibly represented, such as the ad hoc ‘sense impression’ category. For the sense categories of ‘*cold things*’ or ‘*heavy things*,’ Smith *et al*. [42] presented evidence for shifting benefits for both fluency (Study 2 and Study 3) and response novelty (Study 2 only). By contrast, we found that shifting was not beneficial for either fluency or originality on the Sensory Generation Task.

However, the comparison with the earlier study by Smith *et al*. [42] needs to be caveated in two ways. First, and perhaps most notably, our experimental paradigm assesses self-guided or autonomously chosen shifting whereas the paradigm used by Smith and colleagues involved *externally prompted* shifting. Specifically, in the Smith *et al*. [42] paradigm, participants were required to shift between two items at regular, 1 min, intervals. Performance in this (externally prompted) condition was contrasted with a between-subjects condition in which participants worked continuously on one item and were not allowed to shift until after 3 min, after which they worked continuously on the second item. There are many factors that may differ between self-guided and externally prompted switching. Second, in our study the Sensory Generation Task was always the last completed task.

Given that, in the current study, each participant completed both divergent thinking tasks and convergent tasks, and that participants shifted significantly more often for the divergent than convergent tasks (as shown in a significantly higher proportion shift-counts), did this tendency—perhaps indicative of enhanced exploration—carry over to a later convergent task (where it might not be beneficial)? Conversely, and by analogy to research on across-task priming of attentional cognitive activity based on the sparseness versus richness of spatial-foraging [20,46] (but compare also with [23]), did participants' reduced tendency to shift for the convergent tasks carry over to a subsequent divergent task, attenuating how often they shifted between items on the divergent task?

Although not a primary focus of the current study, given that the experimental design also partially manipulated the *order* of the two task types (that is, participants either completed the two convergent tasks first or the two divergent tasks first), we can also examine if there was a main effect of task order, or an interaction of task order with task type, on the shift-count and dwell-length measures. As shown in electronic supplementary material, table S11, there was a significant pattern of *attenuated shifting* on the Alternative Uses Task (the first of the divergent thinking tasks in the divergent mini-block) if it followed the mini-block of convergent tasks (significant task order × task type interaction, cf. [78]). By contrast, there was no carryover from the divergent thinking mini-block to the convergent tasks. Future research might examine carryover effects of task type more systematically by strictly alternating convergent and divergent tasks rather than presenting them in mini-blocks.

Some limitations and additional open questions raised by the current research should be noted. First, aligned with the author’s focal interest in the role of flexibility versus persistence specifically during creative ideation and problem-solving, all of the SGT tasks employed in both the pilot and main study were *idea generation tasks*. All of the tasks required participants to themselves independently produce and report (write) responses that met the given task requirements.

On the one hand, that there were marked and significant within-individual (‘trait-like’) consistencies, across the varied conceptually prompted and perceptually prompted idea generation tasks, in both the process-based measure of flexibility (shift-count) and of persistence (dwell-length) is remarkable, and newsworthy—particularly given the fundamental nature of the question of whether there exists generalized (across-task) consistency in exploration tendencies, and the mixed evidence for such trait-like consistency reviewed in §1. On the other hand, from a broader (higher level) perspective, these same characteristics—that all of the tasks were idea generation tasks, administered under timed conditions, and where the ‘rewards’ or ‘resources’ to be found via search were *participants' own internally generated ideas—*may point to a further boundary condition on when trait-like flexibility versus persistence in behavioural outcome measures may be observed. From this broader perspective, we might ask: is there an important difference between search that is (i) directed internally, towards resources that are only successfully ‘found’ (actually *retrieved*, *made* or *constructed*) by one’s own cognitive-motivational endeavours, versus search that is (ii) directed externally, towards resources that can *only* be ‘found’ (not made), where one’s only possible contribution may be to choose one option or another, and where the outcomes of that choice are entirely dependent on external factors beyond one’s control (e.g. a random, perhaps randomly and dynamically changing reward allocation)?

Despite experimental evidence that points towards the domain-generality of internally directed versus externally directed search [20–22] there is also conflicting evidence [23]. Furthermore, many questions and challenges remain concerning whether indices of exploitation and exploration should be based on observed *behavioural patterns* (switching between options versus remaining at an option, as in the switch-count versus dwell-length measures afforded by the SGT paradigm) versus the *values and uncertainty* associated with choice options (low subjective values and/or high uncertainty versus high subjective values and/or low uncertainty), or the *outcomes obtained from a choice* (information versus rewards) [79]. Crucially, in the context of the idea generation tasks used in the current work, although there were no explicit extrinsic rewards for any of the SGT tasks, participants' success at the task (e.g. imaginatively generating yet another unconventional use for a cup, or spotting a thus-far-overlooked Anagram solution) might itself be intrinsically rewarding [79,80].

The interrelations between successful idea generation, the SGT *process-based* (behavioural) measures of shift-count versus dwell-length, and more semantic *content-based* measures of switching versus clustering likewise remain to be further explored. A not-inconsiderable challenge for such *content-based* measures is that, as Lundin & colleagues [81] recently observed, there is ‘not yet a gold standard for when (or whether) switches occur during memory search’ [81, p. 9] and, furthermore, despite alternative proposals (e.g. linguistic computational similarity models, or participant-based rather than researcher-determined reports) it is not clear if the various plausible approaches should necessarily yield concordant results.

Notably, however, an extensive neurocognitive investigation by Ovando-Tellez *et al*. [82], that used a novel polysemous word-association task and new (multi-variable) integrative measures of clustering versus switching in semantic memory, recently reached conclusions not that different from the main findings outlined here. Circumventing many of the issues related to researcher-defined categories for determining switching versus clustering (noted in §1) via their newly introduced polysemous word-association task, Ovando-Tellez *et al*. [82] defined switches as points at which their (French-speaking) participants' word associations indicated the introduction of a different meaning for each of three different (ambiguous) polysemous words. For example, according to a French linguistic resource for research (Centre National de Ressources Textuelles et Lexicales), the word ‘*Rayon*’ in French has 11 different meanings including, among others, *radius, shelf, wheel spoke, honeycomb and joie, light*. The researchers then computed, for each participant, measures of (i) fluency (the total number of different words generated during the allotted time); (ii) the number of different meanings the responses referred to; (iii) the number of switches between meanings, quantified as the number of times two successive words referred to two different meanings; (iv) the biggest cluster size, defined as the largest number of successive responses that referred to the same meaning during the task; and (v) the response output rank of the first switch, that is the rank of the first response when the participant referred to a new meaning. They then used principal component analyses on the correlational matrix for these five measures, and identified two components. One component captured three of the dependent measures including fluency, biggest cluster size and the output rank of the first switch; they viewed this component as likely reflecting *clustering* of responses within semantic meanings. The second component included the remaining two dependent measures of the number of different meanings and the number of transitions (switches) between meanings; they viewed this component as likely reflecting *switching-related* processes. The two components were not correlated (Spearman *r* = −0.153) and together explained 83% of the variance. All participants also completed several additional cognitive-behavioural tasks including—of especial interest here—a different semantic relatedness judgement task administered during neuroimaging (fMRI) that was used to obtain an individually derived neurocognitive semantic network (‘SemNet’) for each participant.

Contrary to the expectations of Ovando-Tellez *et al*. [82], the clustering component did not seem to reflect spontaneous or automatic semantic associations based on the individually derived semantic network metrics from the relatedness judgement task. Rather, clustering was predicted by higher connectivity between several large-scale brain networks, including the *executive control network* (e.g. the intraparietal sulcus), the *dorsal attention network* (e.g. the superior parietal lobule), as well as the salience and visual networks, with comparatively little connectivity within these networks. By contrast, higher switching was predicted by higher connectivity between the *default mode network* and the *executive control network*, with the default mode network closely involved, as well as higher connectivity between the executive control network, dorsal attention, salience, somatomotor and visual networks.

Given these and related findings—in broad concurrence with the main findings presented herein but that are based exclusively on process-based rather than content-based assessments of flexibility and persistence—Ovando-Tellez *et al*. [82, p. 9] concluded that ‘staying within a cluster may require a focused, sustained, goal-directed attention to suppress interference proactively ... our clustering component may capture ... the tendency to persist longer in a local/exploitation mode.’ They further suggested that ‘the switching component may partly align with the flexibility pathway of the dual-process model of creativity that allows controlled access to broad exploration ... whereas the clustering component may align with the effortful persistence pathway that provides inhibitory focus necessary for systematic thinking’ [82, p. 9].

Second, although in the current study the central findings for shift-count, dwell-length and proportion shift-count strongly replicated between the preliminary data and the preregistered main experiment, the differential outcome for the Anagram task in the main experiment—where each of these SGT process measures for Anagram showed only weak and nonsignificant across-task correlations—was not specifically anticipated. In the pilot study, for *shift-count* across all six pairwise comparisons of the tasks (AUT, FIQ, ANA, STEM), the average correlation (based on Fisher *Zr* transformation) was Fisher *Zr* = .55, with five of the six pairwise comparisons (including two involving ANA) each individually statistically significant (see table 4). The corresponding average correlation for *shift-count* in the main experiment for the six pairwise comparisons of the tasks (AUT, FIQ, STEM, SENSE, excluding ANA) was Fisher *Zr* = .39, with each of the six pairwise comparisons individually statistically significant (five comparisons at *p* < .001, and the remaining comparison at *p* < .01, see table 11). Given our pre-registered hypotheses (§3.1.1, Research Question 1), specifically that, ‘we expect to observe moderate positive pairwise correlations of the measures of shift-count across the SGT tasks [...] we also expect that most of these pairwise correlations will be separately statistically significant’ we did not correct these outcomes for multiple comparisons. Additionally, based on the preliminary data and cognitive task analyses detailed in §1, the pre-registered hypotheses specifically anticipated higher *proportion shift-count* for the two divergent thinking tasks (AUT and FIQ) than for the two predominantly convergent thinking tasks (ANA and STEM). This predicted pattern was clearly observed, with each of the four pairwise comparisons of the average proportion shift-count for these tasks (that is, AUT > ANA, AUT > STEM, FIQ > ANA and FIQ > STEM) statistically significant (all *p* < .001, table 14). Nonetheless, particularly given the differential outcome for the Anagram task in the main experiment, conceptual replication and extension of these findings would be valuable.

Third, it should also be acknowledged that participants in the current study were all students taking part for university course credit, were predominantly in their late teens, and the majority were female. The extent to which the findings might generalize to older or non-student samples, or to problem-solving tasks beyond the five different cognitive search tasks adopted herein, is not known.

A final open question concerns the potential role of other situational or contextual factors, beyond task type, such as an individual’s current (state) level of positive or negative affect and arousal level, or changes in momentary reward prospects [83], on dwell-length and shift-count in Self-Guided Transition tasks. Based on other evidence suggesting that positive affect may modulate cognitive control in the direction of more flexible behaviour [9,84], it might be anticipated that a more positive mood state would be associated with more frequent transitions between items (that is, higher shift-count). This might especially be anticipated for the divergent-thinking tasks, but potentially also (and perhaps detrimentally) for the convergent-thinking tasks that would benefit from maintained goal-pursuit [9]. However, recent work [85] using a spatial search-related foraging paradigm and a mood induction procedure found that whereas *increases* in arousal (relative to the pre-mood induction) were associated with increases in exploratory behaviour, increases in valence (relative to the pre-mood induction) were instead associated with increases in *exploitative* behaviour. Future research might examine how participants' naturally preexisting or experimentally manipulated affective state—prior to engaging in the Self-Guided Transition tasks—modulates shifting and dwelling in divergent versus convergent thinking tasks.

## 7. Conclusion

Using a Self-Guided Transitions paradigm, in which participants are allowed to autonomously choose whether to continue, to switch or to return to working on either of two concurrently presented problem-solving items, the current study provides evidence for both clear within-individual consistency in the proclivity towards flexibility versus persistence, and adaptive modulation of flexibility versus persistence depending on the extent to which the task at hand predominantly calls on divergent versus convergent idea search. These results provide strong support for the presence of an interindividual (trait-like) *across-task* propensity in favour of either flexibility or persistence, and also *task- or context-related modulation* of flexibility. Both shifting and dwelling were associated with the generation of more numerous and more original ideas on divergent-thinking tasks—underscoring the creative and ideational rewards to be found by *both* sometimes staying the course (persistence) and sometimes choosing to shift our focus and efforts in a different direction (flexibility).

## Data Availability

The main study reported here was preregistered on the Open Science Framework, as ‘Self-Guided Transitions, Metacontrol, and Creativity’ (https://osf.io/gzh86). The research followed the preregistered sample size, data exclusions and measures. The data for the study are publicly available [86]. Supplementary material is available online [87].
